# TAZ reverses the inhibitory effects of LPS on the osteogenic differentiation of human periodontal ligament stem cells through the NF-κB signaling pathway

**DOI:** 10.1186/s12903-024-04497-y

**Published:** 2024-06-26

**Authors:** Shuyi Dong, Linglu Jia, Shaoqing Sun, Xingyao Hao, Xiaomei Feng, Yunge Qiu, Ke Gu, Yong Wen

**Affiliations:** 1 School and Hospital of Stomatology, Cheeloo College of Medicine, Shandong University, Jinan, Shandong China; 2 Shandong Key Laboratory of Oral Tissue Regeneration & Shandong Engineering Research Center of Dental Materials and Oral Tissue Regeneration, Jinan, Shandong China; 3 Shandong Provincial Clinical Research Center for Oral Diseases, No.44-1 Wenhua Road West, Jinan, 250012 Shandong China

**Keywords:** TAZ, LPS, Human periodontal ligament stem cells, Osteogenic differentiation, NF-κB pathway

## Abstract

**Background:**

Human periodontal ligament stem cells (hPDLSCs) are important candidate seed cells for periodontal tissue engineering, but the presence of lipopolysaccharide(LPS) in periodontal tissues inhibits the self-renewal and osteogenic differentiation of hPDLSCs. Our previous studies demonstrated that TAZ is a positive regulator of osteogenic differentiation of hPDLSCs, but whether TAZ can protect hPDLSCs from LPS is still unknown. The present study aimed to explore the regulatory effect of TAZ on the osteogenic differentiation of hPDLSCs in an LPS-induced inflammatory model, and to preliminarily reveal the molecular mechanisms related to the NF-κB signaling pathway.

**Methods:**

LPS was added to the culture medium of hPDLSCs. The influence of LPS on hPDLSC proliferation was analyzed by CCK-8 assays. The effects of LPS on hPDLSC osteogenic differentiation were detected by Alizarin Red staining, ALP staining, Western Blot and qRT-PCR analysis of osteogenesis-related genes. The effects of LPS on the osteogenic differentiation of hPDLSCs with TAZ overexpressed or knocked down via lentivirus were analyzed. NF-κB signaling in hPDLSCs was analyzed by Western Blot and immunofluorescence.

**Results:**

LPS inhibited the osteogenic differentiation of hPDLSCs, inhibited TAZ expression, and activated the NF-κB signaling pathway. Overexpressing TAZ in hPDLSCs partly reversed the negative effects of LPS on osteogenic differentiation and inhibited the activation of the NF-κB pathway by LPS. TAZ knockdown enhanced the inhibitory effects of LPS on osteogenesis.

**Conclusion:**

Overexpressing TAZ could partly reverse the inhibitory effects of LPS on the osteogenic differentiation of hPDLSCs, possibly through inhibiting the NF-κB signaling pathway. TAZ is a potential target for improving hPDLSC-based periodontal tissue regeneration in inflammatory environments.

**Supplementary Information:**

The online version contains supplementary material available at 10.1186/s12903-024-04497-y.

## Background

Periodontitis, a widespread inflammatory disease of periodontal tissues, is regarded as one of the most common causes of tooth loss because it generally leads to damage and loss of alveolar bone [1]. Recently, the use of periodontal tissue engineering as a new method for regenerating damaged alveolar bone has attracted increasing attention. Human periodontal ligament stem cells (hPDLSCs), which are present in periodontal tissue, are pluripotent cells that have self-renewal ability and can differentiate into bone, fat, nerve and cartilage [2]. An increasing number of studies indicate that hPDLSCs can be used as “seed cells” for periodontal tissue engineering and can effectively promote the regeneration of periodontal tissues [3].

Notably, there are several microbial components, such as lipopolysaccharide (LPS), in local periodontal tissues of patients with periodontitis. LPS is a class of highly active pathogenic substances unique to gram-negative bacteria, resulting in the infiltration of monocytes, lymphocytes and neutrophils in periodontal tissues, which plays a key role in a range of inflammatory responses and progressive periodontal tissue destruction [4, 5]. When periodontal tissue engineering procedures are applied in patients with periodontitis, residual LPS in the local environment may have adverse effects on hPDLSCs, such as inhibiting the proliferative activity and osteogenic differentiation activity of these cells suppressing hPDLSC-based alveolar bone regeneration [6]. Enhancing the resistance of hPDLSCs to the adverse effects of LPS is highly important.

Transcriptional coactivator with PDZ-binding motif (TAZ) and Yes-associated protein (YAP) are the key effector molecules of the Hippo signaling pathway and have been reported to regulate the functions of stem cells [7]. Several studies have shown that TAZ/YAP have positive effects on the osteogenic differentiation abilities of stem cells. For example, Huang reported that the nuclear translocation of TAZ determined the promotion effects of cytokine Bone morphogenetic protein 9 on osteogenic differentiation of mesenchymal stem cell line C3H10T1/2 [8]; additionally, a hyaluronan-based gel promoted human dental pulp stem cell bone differentiation by activating the TAZ/YAP Pathway [9]. However, a few studies have shown that TAZ/YAP may have detrimental effects on the osteogenic differentiation of stem cells or osteoblasts [10, 11]. Thus, TAZ/YAP may play dual roles in the osteogenic differentiation of stem cells or osteoblasts, depending on the cell type, differentiation timing and so on. Our previous studies revealed that overexpressing TAZ could enhance the osteogenic differentiation potential of hPDLSCs [12, 13], but whether TAZ exerts similar positive effects on hPDLSCs stimulated with LPS is still unknown.

Interestingly, several scholars have proposed that TAZ can affect the activation of the nuclear factor kappa B (NF-κB) signaling pathway [14, 15]. The NF-κB signaling pathway has been shown to be closely related to the effects of LPS on cell functions [16]: LPS binds to the corresponding receptors on cells, subsequently causing a series of protein and kinase reactions leading to the phosphorylation of IκBα, followed by the activation and nuclear translocation of P50/P65 dimers (NF-κB); subsequently, the transcription of a series of downstream target genes is promoted [17, 18]. However, the effects of TAZ on NF-κB signaling seem controversial: TAZ has been proven to inhibit the transduction of the NF-κB signaling pathway in several types of cells, such as microglia [19], osteoclasts [20], and alveolar epithelial type II cells [15], while a study in endothelial cells showed that knockdown of TAZ/YAP did not affect NF-κB signaling induced by TNF-α [21]. Another study reported that TAZ/YAP acted as a positive regulator of the transduction of the NF-κB signaling pathway in breast cancer [22]. Therefore, the role of TAZ in the transduction of the NF-κB signaling pathway is still unclear, and its regulatory role in hPDLSCs is still unknown.

The purpose of the present study was to explore whether TAZ could alleviate the inhibitory effects of LPS on the osteogenic differentiation of hPDLSCs and to preliminarily explore the molecular mechanisms related to the NF-κB signaling pathway.

## Methods

### Isolation, cultivation and identification of hPDLSCs

After volunteers (6 volunteers, aged 16–24, no systemic diseases) at Shandong University Stomatological Hospital signed informed consent forms, their premolars extracted for orthodontic treatment were collected. All procedures for collecting isolated teeth were performed in accordance with procedures approved by the Ethics Committee of Shandong University Stomatological Hospital. The middle 1/3 of the periodontal tissues on the root surface of the teeth were collected, chopped and inoculated on the bottom of a culture bottle. After the culture bottle was put in a 37 °C incubator for approximately 2–4 h, the tissues firmly adhered to the bottom of the culture bottle, then they were cultured in the primary culture medium (containing 20% FBS (IONSERA, Uruguay) + α-MEM (Basalmedia, China) + 10% penicillin and streptomycin solution (Beyotime, China)) for 7–14 days. Cells were passaged after they reached 90% confluence, and cells at passages 3–5 were used for the experiments.

The immunophenotype of the hPDLSCs was analyzed by a BD Stemflow™ hMSC Analysis Kit (#562,245, BD, USA), and all procedures followed the manufacturer’s instructions. Briefly, 5 × 10^6^ cells/mL were prepared and incubated in stain buffer containing CD44, CD90, CD105, CD34, or CD45 antibodies which were diluted according to the instructions, then the cells were analyzed by a flow cytometry.

The hPDLSCs were seeded in a 12-well tissue culture plate at a density of 10^5^ cells/well, then were cultured in the adipogenic medium (complete culture medium (10% FBS + α-MEM) supplemented with 1 µM dexamethasone (Solarbio, China), 0.2 mM indomethacin (Solarbio), 0.01 g/l insulin (Solarbio) and 0.5 mM isobutyl-methylxanthine (Solarbio)) for 14 days and then stained with Oil Red O.

The hPDLSCs were seeded in a 12-well tissue culture plate at a density of 10^5^ cells/well, then were cultured in the osteogenic medium (complete culture medium supplemented with 10 nM dexamethasone (Solarbio), 10 mM β-glycerophosphate (Solarbio) and 50 mg/l ascorbic acid (Solarbio)) for 14 days and then were stained with Alizarin Red.

In colony-formation assay, the hPDLSCs were inoculated into a culture dish at a density of 10 cell/mL, then were cultured for 14 days and stained with crystal violet.

### Cell treatments with LPS

The hPDLSCs were cultured in complete culture medium supplemented with different concentrations of LPS (Sigma-Aldrich, USA) (0.1–100 µg/mL) and then were subjected to Cell Counting Kit-8 (CCk-8) assays, Western Blot assays, qRT-PCR assays, and Immunofluorescence staining.

### CCK-8

The cells were seeded in a 96-well tissue culture plate at a density of 3 × 10^3^ cells/well and were continuously cultured with complete culture medium for 2, 4, or 6 days. At each time point, cell proliferation activity was analyzed by a CCK-8 kit (Dojindo Laboratories, Japan) according to the manufacturer’s instructions. Briefly, the culture medium in each well was replaced with 10 µL of cell counting solution + 90 µL of blank medium, and the plates were then incubated in an incubator for 2 h. The absorbance at 450 nm was measured with a microplate reader.

### Alkaline phosphatase (ALP) staining and Alizarin Red staining

The cells were seeded in a 12-well tissue culture plate at a density of 10^5^ cells/well and were continuously cultured with osteogenic induction medium. When necessary, 10 µg/mL LPS was added into the medium. After 14 or 21 days, the cells were washed with phosphate buffer solution (PBS) and then fixed with 4% paraformaldehyde for 30 min. ALP staining solution (NBT/BCIP staining kit, Beyotime, China) or Alizarin Red staining solution (Solarbio) were used according to the manufacturer’s instructions.

The full-field photo of cells with ALP staining and Alizarin Red staining in each well was taken by a camera, so the staining intensity of different groups was compared directly. In addition, 3 random fields of view in each well were observed and taken photos under a microscope to further compare the staining intensity between different groups.

### Protein isolation and Western Blot analysis

Cells were lysed to extract proteins using RIPA buffer (Solarbio) supplemented with PMSF (Solarbio) and proteinase and phosphatase inhibitor cocktail (Bosterbio, USA) according to the manufacturer’s instructions. Then, the protein concentration was determined by the BCA kit (Solarbio), and finally, protein denaturation was performed in 100℃ for 5 min with SDS-PAGE loading buffer (Beyotime). The 10% separating gel and 4% stacking gel were prepared according to standard methods (Beyotime). After the sample was loaded at a certain concentration, the gel was subjected to electrophoresis (Bio-Rad, USA). The proteins in the gel were transferred to a polyvinylidene fluoride (PVDF) membrane (Millipore, USA), blocked with milk powder (Solarbio), and incubated with primary antibodies overnight at 4℃. After washing with TBST buffer, the membranes were incubated with the appropriate secondary antibody at room temperature for 1 h. Finally, the protein bands were observed using an automatic fluorescence imaging analyzer (TIANNENG, China) with enhanced chemiluminescent ECL reagent (Millipore, USA). The grayscale values of protein bands were analyzed by image J software.

Primary and secondary antibodies included: p-P65 (#3033 rabbit mAb, CST, USA, 1:1000), P65 (#8242 rabbit mAb, CST, 1:1000), p-IκBα (#2859 rabbit mAb, CST, 1:1000), IκBα(#4814 mouse mAb, CST, 1:1000), RUNX2 (#8486 rabbit mAb, CST, 1:1000), TAZ (#4883 rabbit mAb, CST, 1:1000), GAPDH (#5174 rabbit mAb, CST, 1:1000), ALP(11187-1-AP, rabbit mAb, Proteintech, China, 1:1000), COL1(#0088, rabbit mAb, Wanleibio, China, 1:1000), Anti-mouse IgG HRP-linked Antibody (#7076, horse antibody, CST, 1:2000), and Anti-rabbit IgG HRP-linked Antibody (#7074, goat antibody, CST, 1:2000).

### RNA isolation and qRT-PCR

The AG RNAex Pro Reagent (Accurate Biology, China) was used to extract the total RNA of the cells. After the addition of chloroform and centrifugation, the supernatant layer was collected, and the total RNA was precipitated with isopropanol. cDNA was obtained using a reverse transcription kit (Evo M-MLV RT Kit with gDNA Clean for qRT-PCR II AG11711, Accurate Biology) for qRT-PCR detection. The qRT-PCR program was: 95 °C for 15 s, followed by 45 cycles at 60 °C for 60 s, and 72 °C for 60 s in a LightCycler® 480II machine (Roche, Switzerland). The sequences of the primers used for qRT-PCR are shown in Table 1 (Sangon Biotech, China). The gene expression of all samples in different groups was normalized to that of GAPDH and calculated by the 2^‑ΔΔCT^ method.

**Table 1:** Primers for qRT-PCR

Genes	Forward primer 5′-3′	Reverse primer 5′-3
RUNX2	GTTTCACCTTGACCATAACCGT	GGGACACCTACTCTCATACTGG
Col1	GCTGATGATGCCAATGTGGTT	CCAGTCAGAGTGGCACATCTTG
OCN	AATCCGGACTGTGACGAGTTG	CAGCAGAGCGACACCCTAGAC
Il-6	AGCCCACCGGGAACGA	GGACCGAAGGCGCTTGT
Il-8	TTGGCAGCCTTCCTGATTTC	AACTTCTCCACAACCCTCTGCA
TAZ	GAGAGTACATGAACCTGA	ATCAACTTCAGGTTCCAG
GAPDH	AGAAGGCTGGGGCTGAGA	AGGGGCCATCCACAGTCT

### Immunofluorescence staining

The hPDLSCs were seeded in 24-well plates (5 × 10^4^ cells/well) and treated with LPS (10 µg/ml) for 24 h. The cells were fixed with 4% paraformaldehyde for 30 min, washed 3 times with PBS, and then incubated with 0.5% TritonX-100 for 10 min. The cells were incubated with primary antibodies was at 4°C overnight and then incubated with fluorescent secondary antibodies at room temperature for one hour. After being stained with DAPI (Solarbio) for 10 min, the cells were observed under a fluorescence microscope (Leica, Germany). 3 random fields of view in each well were photographed to compare the distribution of P65 protein in cells between different groups.

Primary and secondary antibodies included: P65 (#8242 rabbit mAb, CST, 1:500), Goat Anti-Rabbit IgG/RBITC (SR134, Solarbio, 1:200), and Goat anti-Rabbit IgG/FITC (SF134, Solarbio, 1:200).

### Lentivirus transfection assay

TAZ-overexpressing (oeTAZ), TAZ-knockdown (shTAZ), and their corresponding empty vector lentivirus (oeNC, shNC) were constructed by Shanghai GenePhaema company (China). Then, 4 × 10^5^ hPDLSCs at the third passage were seeded in a medium dish (diameter 6 cm) and treated with TAZ-overexpressing or TAZ-knockdown lentivirus and their corresponding empty vector lentivirus. After 12 h of transfection, the medium was replaced with culture medium containing puromycin (Beyotime). Finally, the knockdown or overexpression efficiency was verified by Western Blot and qRT-PCR analyses.

### Statistical analysis

All experiments were repeated at least three times independently. The results are expressed as the mean ± SD. The experimental data were analyzed for normality test and variance homogeneity test, and student’s t test or one-way ANOVA was used to analyze significant differences between groups. *P* < 0.05 indicated a significant difference. GraphPad Prism 8.0 software was used for the statistical analyses.

## Results

### Characterization of hPDLSCs

**Fig. 1:**
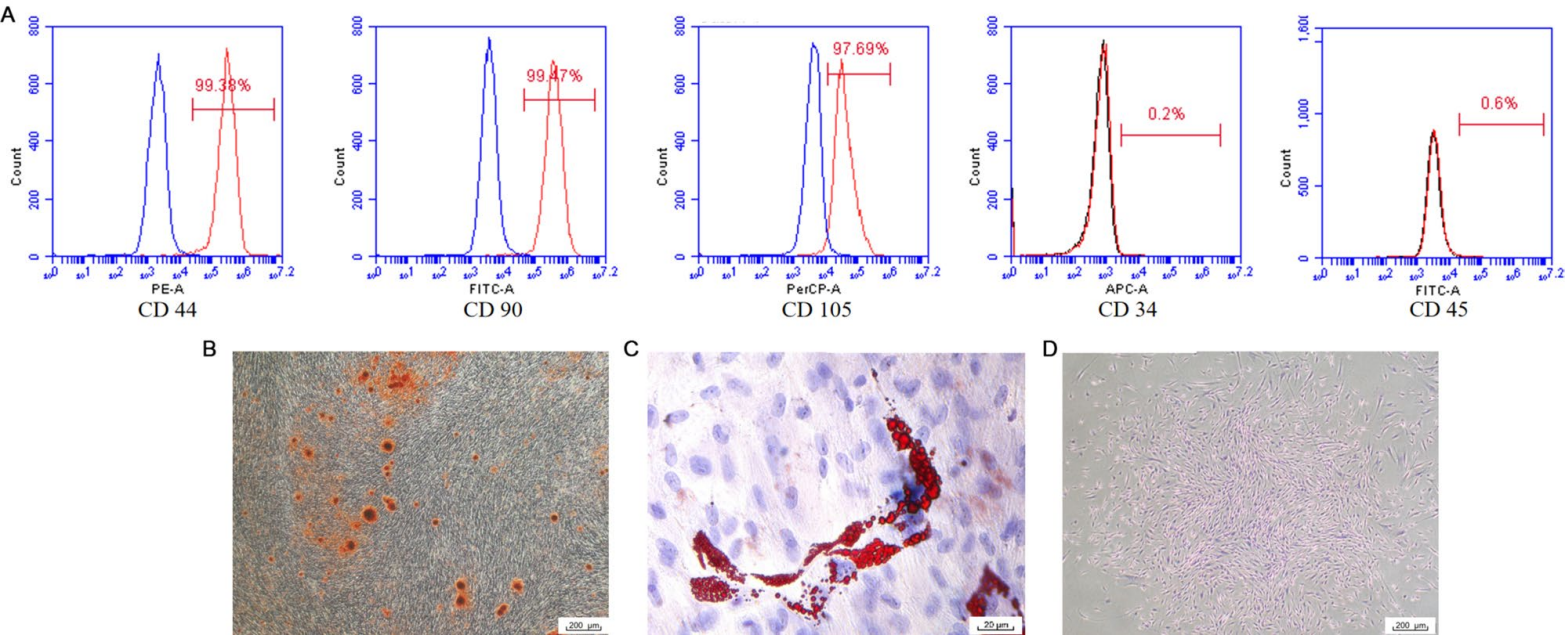
Characterization of hPDLSCs. (**A**) Flow cytometry analysis indicated that hPDLSCs were positive for CD44, CD90, and CD105, and negative for CD34 and CD45. (**B**) Red calcium deposits were observed in hPDLSCs after staining with alizarin red. (**C**) Red lipid droplets were observed in hPDLSCs after oil red O staining. (**D**) Cell colonies were observed after staining with crystal violet

Flow cytometry analyses revealed that the hPDLSCs were positive for mesenchymal stem cell-specific surface markers such as CD44, CD90, and CD105 and negative for endothelial cell-specific markers such as CD34 and CD45, indicating that the hPDLSCs exhibited the characteristics of mesenchymal stem cells (Fig. 1A). After osteogenesis or lipogenesis induction for 14 days, obvious red mineralized nodules or red lipid droplets were observed, indicating that the hPDLSCs underwent adipogenic and osteogenic differentiation (Fig. 1B, C). According to the results of the colony formation assay, the hPDLSCs exhibited strong proliferative ability and formed cell colonies (Fig. 1D). All of the above results proved that the hPDLSCs possessed the characteristics of mesenchymal stem cells.

### LPS inhibited the osteogenic differentiation of hPDLSCs

**Fig. 2:**
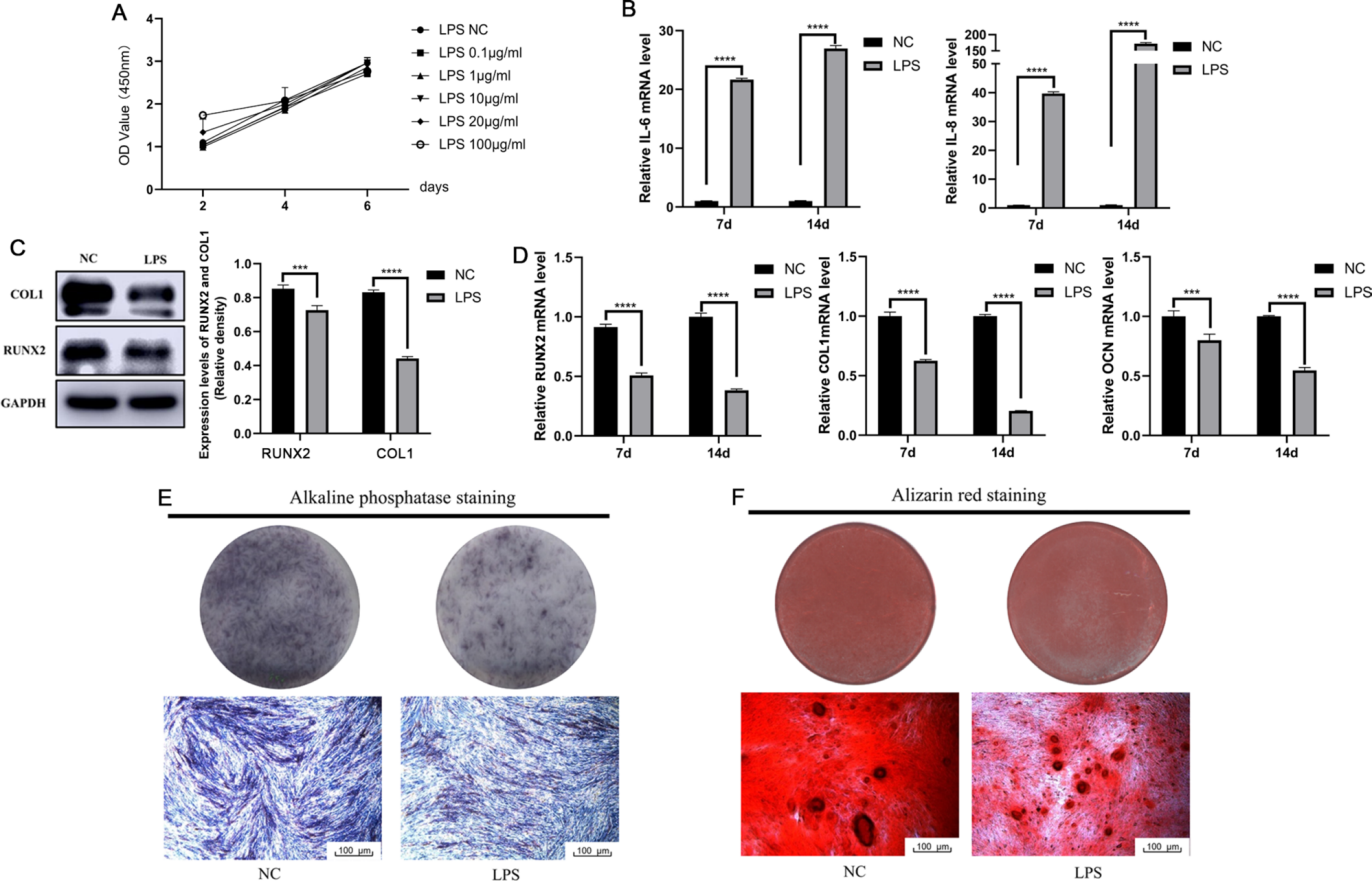
LPS inhibited the osteogenic differentiation of hPDLSCs. (**A**) The CCK-8 results showed that LPS (0.1–10 µg/mL) did not influence the proliferation of hPDLSCs. (**B**) The qRT-PCR results showed that the expression levels of IL-6 and IL-8 were significantly increased after the hPDLSCs were treated with 10 µg/mL LPS for 7d and 14d. (**C**) The Western Blot results showed that the expression level of osteogenesis-related proteins decreased when hPDLSCs were treated with 10 µg/mL LPS during osteogenic induction for 7d. (**D**) The qRT-PCR results showed that the expression level of osteogenesis-related mRNAs decreased when hPDLSCs were treated with 10 µg/mL LPS during osteogenic induction for 7d. (**E**) ALP staining on day 7 when hPDLSCs were treated with 10 µg/mL LPS during osteogenic induction. (**F**) Alizarin Red staining on day 14 when hPDLSCs were treated with 10 µg/ml LPS during osteogenic induction. ****P ≤ 0.0001

After hPDLSCs were stimulated with different concentrations of LPS (0.1–100 µg/mL) for 2, 4, or 6 days, a CCK-8 assay was carried out to analyze the effects of LPS on the proliferation of hPDLSCs. The results showed that all concentrations of LPS had no significant effect on the proliferation of hPDLSCs (Fig. 2A). The qRT-PCR results showed that the expression levels of inflammatory factors such as IL-6 and IL-8 were significantly increased after the hPDLSCs were treated with LPS for 7 days or 14 days (Fig. 2B), suggesting that LPS promoted the release of inflammatory cytokines.

Western Blot and qRT-PCR showed that LPS significantly reduced the protein and mRNA levels of osteogenesis-related genes, including RUNX2 and COL1, in hPDLSCs after osteogenic induction (Fig. 2C, D). In addition, the results of ALP staining showed that the group treated with LPS had lighter staining than the group not treated with LPS, proving that LPS inhibited the ALP activity of hPDLSCs (Fig. 2E). Alizarin Red staining revealed that the amount of reddish-brown staining in the LPS group was significantly weaker than that in the no-LPS group, and the number of mineralized nodules was significantly lower in the LPS group than in the control group, as observed under a microscope (Fig. 2F). These results confirmed that LPS inhibited the osteogenic differentiation of hPDLSCs.

### LPS inhibited the expression of TAZ and promoted the activation of the NF-κB signaling pathway in hPDLSCs

**Fig. 3:**
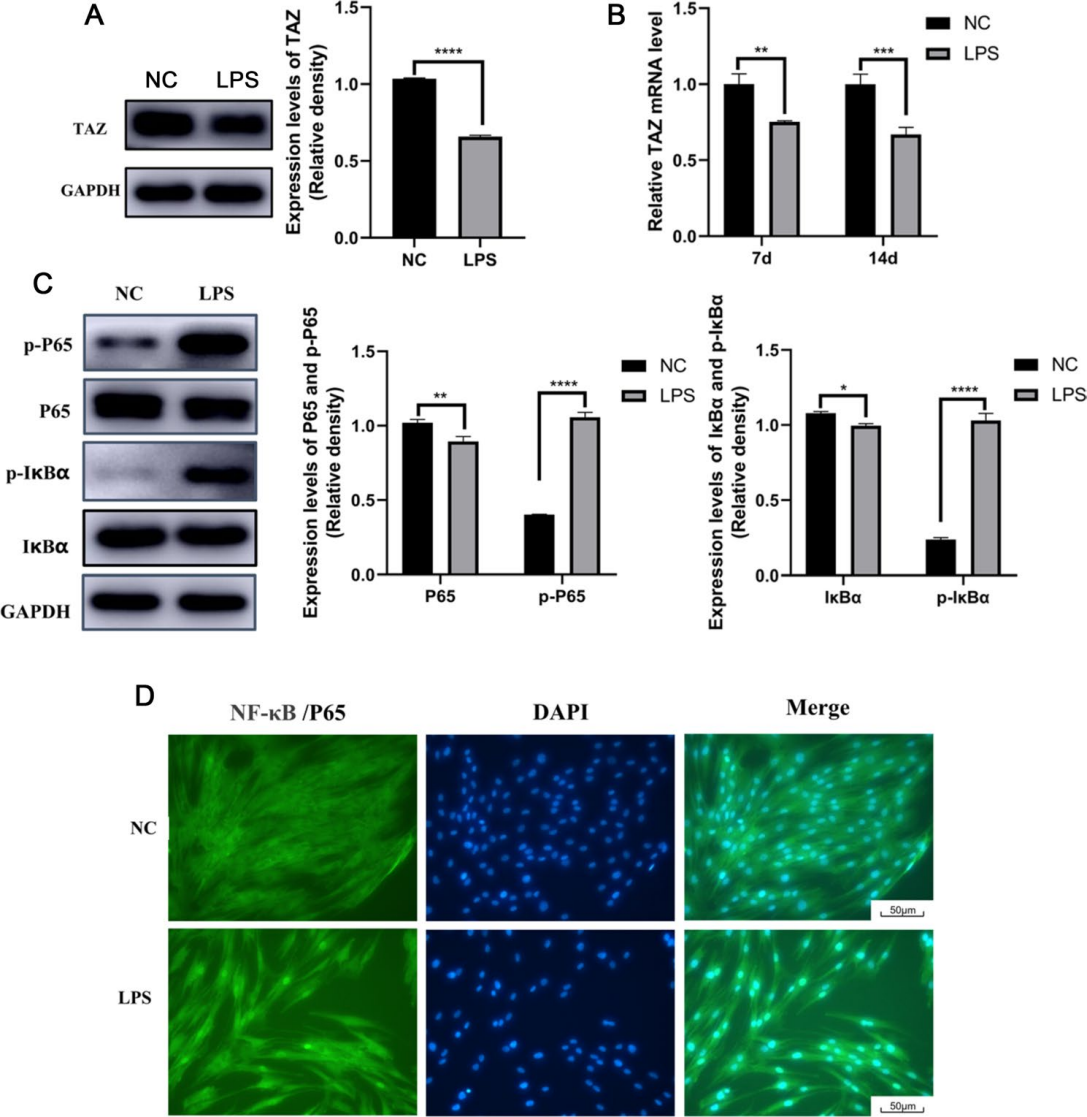
LPS inhibited the expression of TAZ and promoted the activation of the NF-κB signaling pathway in hPDLSCs. (**A**) Western Blot manifested the protein level of TAZ in hPDLSCs stimulated with 10 µg/mL LPS for 24 h. (**B**) qRT-PCR manifested the mRNA level of TAZ in hPDLSCs stimulated with 10 µg/mL LPS for 24 h. (**C**) Western Blot revealed that the p-P65/P65 and p-IκBα/IκBα ratios were significantly increased in hPDLSCs stimulated with 10 µg/mL LPS for 24 h. (**D**) Immunofluorescence analysis revealed that P65 entry into the nucleus was significantly increased when hPDLSCs were stimulated with 10 µg/mL LPS for 24 h. *P ≤ 0.05, **P ≤ 0.01, ***P ≤ 0.001, ****P ≤ 0.0001

TAZ expression in hPDLSCs stimulated with LPS was analyzed. The Western Blot and qRT-PCR results indicated that the expression of TAZ decreased significantly when LPS was added to the culture medium (Fig. 3A, B). The expression level of NF-κB signaling pathway components in hPDLSCs was also analyzed. After the hPDLSCs were treated with LPS for 1 day, Western Blot analysis revealed that the ratios of p-IκBα/IκBα and p-P65/P65 ratios were significantly increased; that is, LPS significantly promoted the activation of IκBα and P65 (Fig. 3C). Moreover, immunofluorescence analysis revealed that P65 entry into the nucleus was significantly increased when the cells were stimulated with LPS, while in the group not stimulated with LPS, almost all of the P65 was located in the cytoplasm (Fig. 3D). These results indicated that LPS inhibited the expression of TAZ and promoted the activation of the NF-κB signaling pathway in hPDLSCs. We speculated that TAZ may be involved in the inhibitory effect of LPS on the osteogenic differentiation of hPDLSCs.

### Efficiency of TAZ overexpression and knockdown in hPDLSCs

Lentivirus transfection was used to specifically knock down or overexpress TAZ in hPDLSCs, and the cells were observed under a fluorescence microscope 48 h after transfection.

**Fig. 4:**
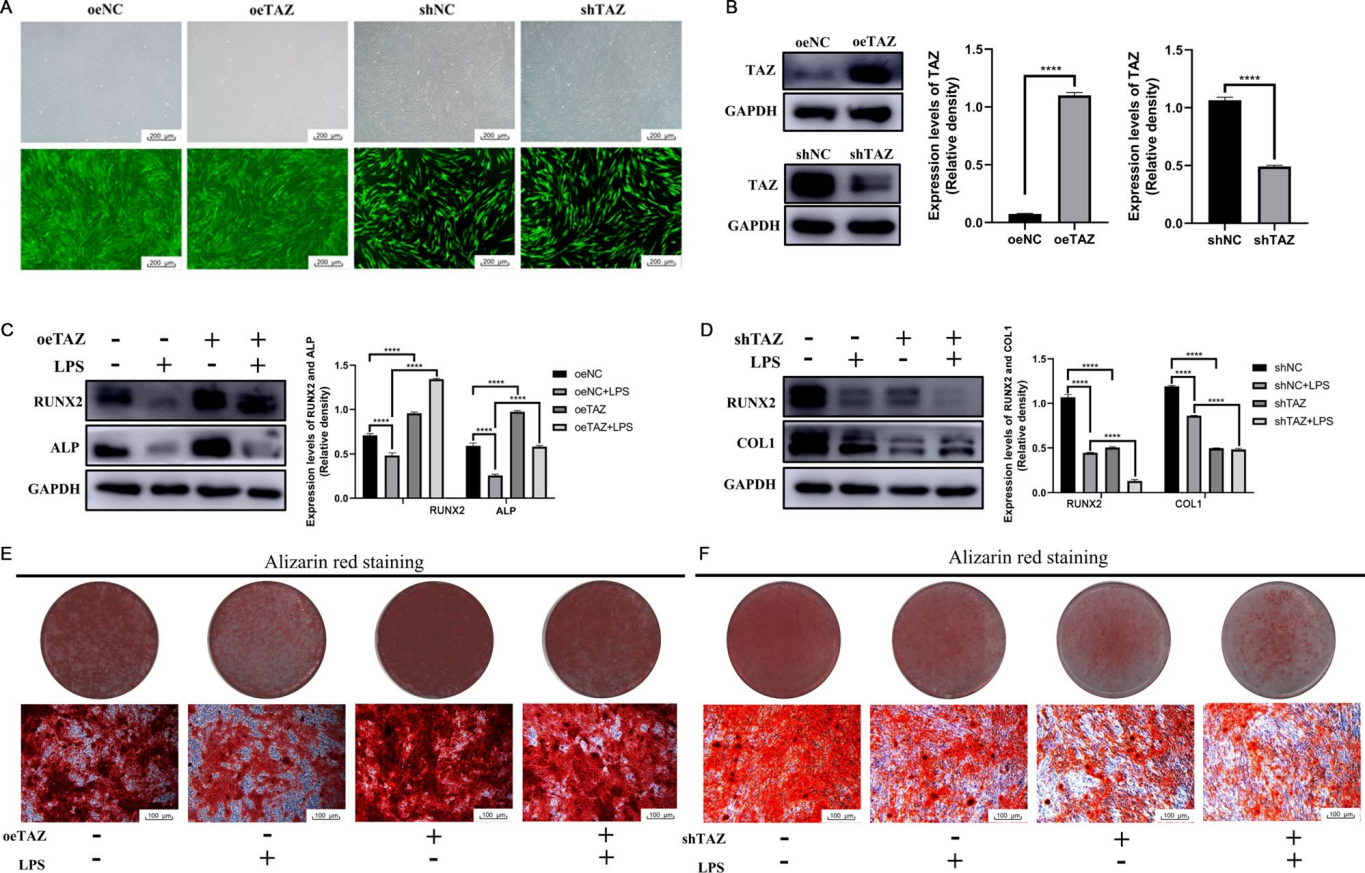
TAZ reversed the inhibitory effect of LPS on the osteogenic differentiation of hPDLSCs. (**A**) The hPDLSCs infected with lentivirus showed green fluorescence under a fluorescence microscope. (**B**) The efficiency of overexpressing and knocking down TAZ in hPDLSCs was analyzed by Western Blot. (**C**) (**D**) Western Blot showed the expressions of osteogenesis-related proteins in the TAZ knockdown group and the TAZ overexpression group with the stimulation of 10 µg/mL LPS. (**E**) (**F**) Scanning and microscopic observation of Alizarin Red staining. shTAZ: hPDLSCs with TAZ knocked down. shNC: the control group for shTAZ hPDLSCs. oeTAZ: hPDLSCs with TAZ overexpressed. oeNC: the control group for oeTAZ hPDLSCs. *P ≤ 0.05, **P ≤ 0.01, ***P ≤ 0.001, ****P ≤ 0.0001

Most of the cells were marked with green fluorescence, and the transfection efficiency was judged to be greater than 80% (Fig. 4A). Western Blot analysis revealed that TAZ expression was significantly decreased in the TAZ knockdown group but significantly increased in the TAZ overexpression group (Fig. 4B). These results indicated that an hPDLSC model with specific knockdown or overexpression of TAZ was successfully constructed.

### TAZ reversed the inhibitory effect of LPS on the osteogenic differentiation of hPDLSCs

Western Blot results showed that the expression of osteoblast-related proteins in the shNC + LPS group was lower than that in the shNC group, and similarly, in the oeNC + LPS group, they were also significantly lower than that in oeNC group (Fig. 4C, D). After TAZ knockdown, the expression of RUNX2 and COL1 in the shTAZ + LPS group was significantly lower than that in the shNC + LPS group (Fig. 4D); after TAZ was overexpressed, the expressions of RUNX2 and ALP in the oeTAZ + LPS group was significantly greater than that in the oeNC + LPS group (Fig. 4C).

**Fig. 5:**
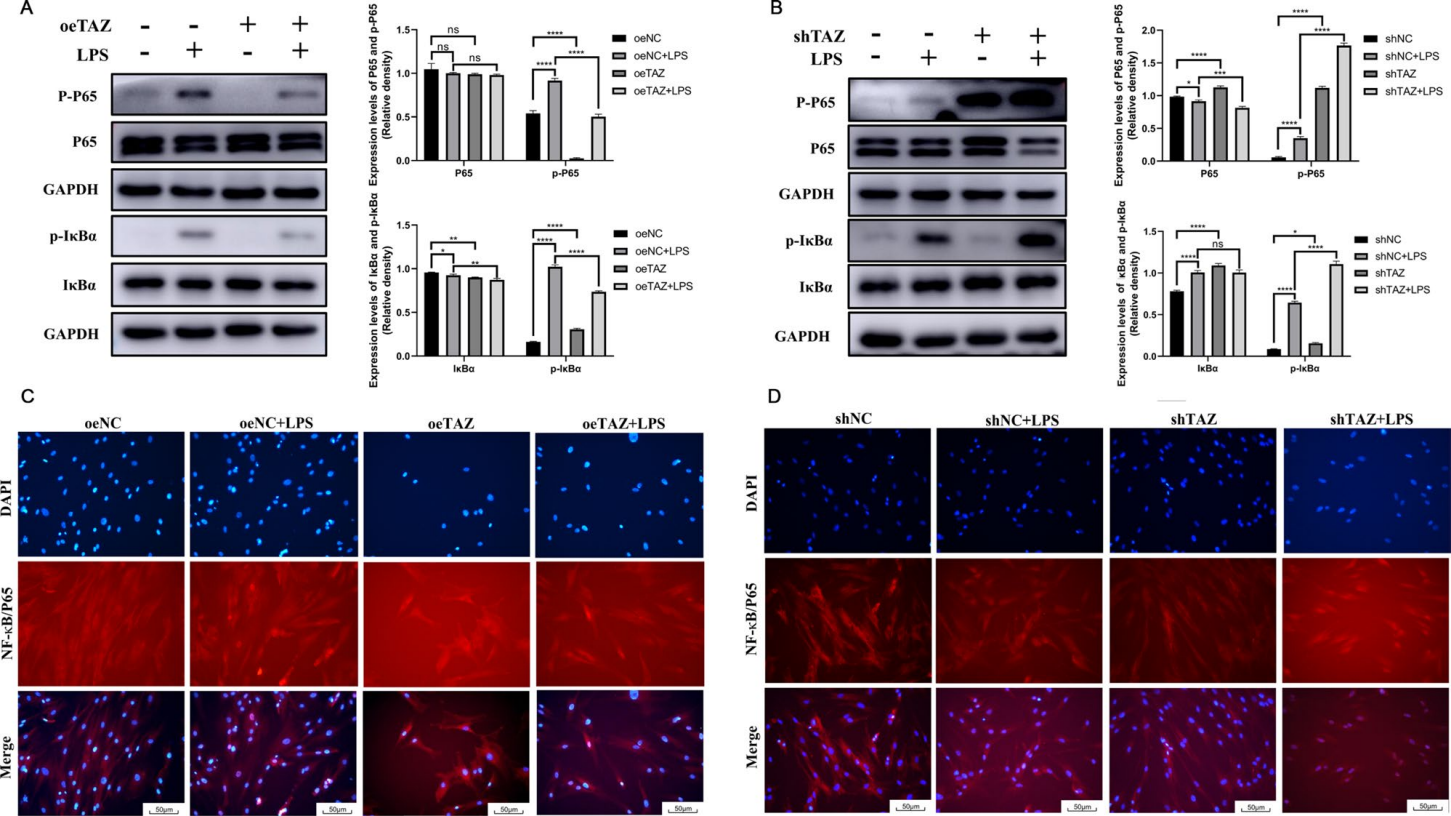
TAZ affected the LPS-induced activation of the NF-κB signaling pathway in hPDLSCs. (**A**) Western Blot showed that overexpressing TAZ inhibited the effects of 10 µg/mL LPS on the phosphorylation of P65 and IκBα. (**B**) Western Blot showed that knocking down TAZ enhanced the effects of 10 µg/mL LPS on the phosphorylation of P65 and IκBα. (**C**) Immunofluorescence showed that overexpressing TAZ inhibited the nuclear translocation of P65 induced by 10 µg/mL LPS. (**D**) Immunofluorescence showed that knocking down TAZ promoted the nuclear translocation of P65 induced by 10 µg/mL LPS. shTAZ: hPDLSCs with TAZ knocked down. shNC: the control group for shTAZ hPDLSCs. oeTAZ: hPDLSCs with TAZ overexpressed. oeNC: the control group for oeTAZ hPDLSCs. *P ≤ 0.05, **P ≤ 0.01, ***P ≤ 0.001, ****P ≤ 0.0001

Alizarin Red staining revealed that the staining in the shNC + LPS group was weaker than that in the shNC group (Fig. 4F), the staining in the oeNC + LPS group was also significantly weaker than that in the oeNC group, and the mineralized nodules were reduced (Fig. 4E). After specific overexpression of TAZ, the staining of the oeTAZ + LPS group was significantly greater than that of the oeNC + LPS group, and the number of mineralized nodules was significantly greater (Fig. 5E). After specific knockdown of TAZ, the staining of the shTAZ + LPS group was weakened compared with that of the shNC + LPS group, and the number of mineralized nodules was significantly reduced (Fig. 5F).

Taken together, these results demonstrated that overexpressing TAZ reversed the inhibitory effect of LPS on the osteogenic differentiation of hPDLSCs, while knocking down TAZ exacerbated the inhibitory effect of LPS on the osteogenic differentiation of hPDLSCs.

### TAZ affected the LPS-induced activation of the NF-κB signaling pathway

The influence of TAZ on the expression of NF-κB signaling pathway components was analyzed. After 1 day of LPS stimulation, the Western Blot results showed that the p-IκBα/IκBα and p-P65/P65 ratios in the oeNC + LPS group were significantly greater than those in the oeNC group (Fig. 5A), and the p-IκBα/IκBα and p-P65/P65 ratios in the shNC + LPS group were also significantly greater than those in the shNC group (Fig. 5B). After specific overexpression of TAZ, the expression of p-IκBα/IκBα and p-P65/P65 in the oeTAZ + LPS group was significantly lower than that in the oeNC + LPS group, suggesting that overexpression of TAZ could significantly inhibit the activation of the NF-κB signaling pathway (Fig. 5A). After specific knockdown of TAZ, the expression of p-IκBα/IκBα and p-P65/P65 in the shTAZ + LPS group was greater than that in the shNC + LPS group, indicating that knockdown of TAZ significantly promoted the activation of the NF-κB signaling pathway (Fig. 5B).

We also detected the localization of the P65 protein within the cell. According to the immunofluorescence results, significantly more P65 entered the nucleus in the oeNC + LPS group than in the oeNC group (Fig. 5C), and significantly more P65 entered the nucleus more in the shNC + LPS group than in the shNC group (Fig. 5D). After specific overexpression of TAZ, the entry of P65 into the nucleus in the oeTAZ + LPS group was significantly reduced compared with that in the oeNC + LPS group (Fig. 5C), indicating that activation of the NF-κB signaling pathway was inhibited to a certain extent. After specific knockdown of TAZ, P65 entry into the nucleus in the shTAZ + LPS group was significantly greater than that in the shNC + LPS group (Fig. 5D), indicating that TAZ knockdown significantly promoted NF-κB signaling pathway activation.

All of the above results proved that TAZ inhibited the activation of the NF-κB signaling pathway, which could be the molecular mechanism by which TAZ reversed the inhibitory effects of LPS on the osteogenic differentiation of hPDLSCs.

## Discussion

In periodontitis, LPS is considered a harmful factor that causes inflammation and cell damage in periodontal tissues. Since the proliferative activity and osteogenic differentiation ability of hPDLSCs are important for hPDLSC-based alveolar bone regeneration, the influence of LPS on hPDLSC proliferation and osteogenesis in vitro was analyzed. Our results showed that LPS at a concentration of 0.1–100 µg/ml did not alter the proliferation trend of hPDLSCs within 6 days, which was consistent with the conclusions of many researchers [23–25]. However, LPS at a concentration of 10 µg/ml significantly inhibited the osteogenic differentiation of hPDLSCs, which was in line with the findings of several previous studies [16, 26, 27]. Notably, the effects of LPS on cell osteogenic differentiation could differ based on the concentration of LPS because several previous studies reported that low concentrations of LPS (less than 1 µg/ml) had a positive effect on the osteogenesis of stem cells in vitro [24, 25, 28–30]. Considering that there may be a large amount of residual LPS in the periodontal tissues of patients with periodontitis, it is still necessary to protect hPDLSCs from the potential negative effects of LPS on osteogenic differentiation.

TAZ is one of the key effector molecules of the Hippo signaling pathway, and our previous study demonstrated that it can affect the proliferation, apoptosis, and osteogenic differentiation of hPDLSCs [13, 31]. The present study showed that the expression level of TAZ decreased when hPDLSCs were treated with LPS, suggesting that the inhibitory effects of LPS on the osteogenic differentiation of hPDLSCs may be related to TAZ. In the following study, overexpression of TAZ partly reversed the negative influence of LPS on the osteogenesis of hPDLSCs. These results proved that targeting TAZ could be a useful method for restoring the osteo-differentiation potential of hPDLSCs in the LPS microenvironment. In fact, as mentioned above, the regulatory effects of TAZ on cell osteogenic differentiation are controversial. Although a considerable portion of research suggests that TAZ is a positive regulator of osteogenesis [8, 9], several studies have shown that TAZ may inhibit the osteogenic process to some extent. For instance, TAZ/YAP were shown to suppress canonical Wnt signaling and Runx2 activity in mouse osteoblast progenitors and thus opposed differentiation toward the osteoblast lineage [11]. It can be inferred that the regulatory effect of TAZ may vary with changes in cell type and microenvironments. Anyway, the present results showed that TAZ could effectively enhance the osteogenic potential of hPDLSCs stimulated by LPS, so overexpressing TAZ could be a potential method to protect the osteogenic differentiation ability of hPDLSCs from LPS. The limitation of the present research is that the experiments were conducted only in vitro, and more in vivo studies are needed to verify the benefits of overexpressing TAZ in periodontitis, which will be our future study directions. Additionally, small molecule activators of TAZ may be useful and convenient for promoting hPDLSC-based alveolar bone regeneration in patients with periodontitis.

To explore the mechanism by which TAZ promotes hPDLSCs osteogenic differentiation under LPS stimulation, our research focused on the NF-κB signaling pathway, a classic inflammation-related signaling pathway. In fact, previous studies have shown that NF-κB plays diverse roles in the regulation of stem cells osteogenesis and is influenced by cell type, organism, and other factors. For example, in human dental pulp stem cells, the activation of NF-κB signaling pathways induced by TNF-α was reported to trigger the osteogenic differentiation of cells [32], while the osteogenic differentiation potential of dental pulp stem cells derived from estrogen-deficient rats decreased, accompanied by elevated NF-κB activity [33]; for hPDLSCs, the activation of the NF-κB pathway reportedly decreased their osteogenic potential [34–37]. Our results suggested that the activation of the NF-κB signaling pathway by LPS had negative effects on the osteogenesis of hPDLSCs. The mechanism of the inhibitory effects of NF-κB on hPDLSCs was probably related with its role of transcription factor, for it promotes the expression of several inflammatory cytokines, such as IL1β and IL6, which could damage the vitality of stem cells and inhibit the osteogenic differentiation of hPDLSCs [38, 39]. Therefore, inhibiting the activation of the NF-κB signaling pathway is meaningful in reducing the damage of LPS on the osteogenic differentiation ability of hPDLSCs.

The present results showed that LPS significantly activated the NF-κB signaling pathway in hPDLSCs, while overexpression of TAZ reduced the phosphorylation of IκBα, thus reducing the activation and nuclear translocation of P65. Therefore, we speculated that TAZ protected the osteogenic differentiation ability of hPDLSCs from the negative effects of LPS by inhibiting NF-κB signaling. Our results are similar to those of several studies on the relationships between TAZ and NF-κB signaling: TAZ ameliorates the inflammatory response in microglia via inhibition of the NF-κB pathway [19]; TAZ inhibits the transduction of the NF-κB signaling pathway in osteoclasts and thus inhibits osteoclastogenesis [20]; TAZ can upregulate IκB and subsequently inhibit NF-κB mediated inflammatory responses in alveolar epithelial type II cells [15]. However, other studies have reported that TAZ does not influence or promote the transduction of NF-κB signals [21, 22]. It can be inferred that the regulatory relationship between TAZ and NF-κB may change due to differences in cell type or microenvironment. Our present results showed that TAZ plays a negative role in the NF-κB signaling pathway, which could be one of the mechanisms by which TAZ protects the osteogenic differentiation of hPDLSCs from LPS. However, we did not explore how TAZ reduced the phosphorylation levels of IκBα, which is a limitation of the present research. We will further study this unresolved issue in our future research and try to reveal the molecular mechanism of TAZ in more detail.

## Conclusion

The present study revealed that TAZ could reverse the inhibitory effects of LPS on the osteogenic differentiation of hPDLSCs, possibly through the NF-κB signaling pathway. TAZ is a potential target for improving hPDLSC-based periodontal tissue regeneration in inflammatory environments.

### Electronic supplementary material

Below is the link to the electronic supplementary material.

Supplementary Material 1

## Data Availability

All data generated or analyzed during this study are included in this published article.

## Abbreviations

ALP: Alkaline phosphatase
CCk-8: Cell-Counting-Kit-8
FBS: Fetal bovine serum
hPDLSCs: human periodontal ligament stem cells
LPS: Lipopolysaccharide
PBS: Phosphate buffer saline
qRT-PCR: quantitative real-time polymerase chain reaction
TAZ: Transcriptional coactivator with PDZ-binding motif
YAP: Yes-associated protein
