# Supplementary material for: TAZ reverses the inhibitory effects of LPS on the osteogenic differentiation of human periodontal ligament stem cells through the NF-κB signaling pathway
Source: BMC Oral Health. 2024 Jun 26;24:733. doi: 10.1186/s12903-024-04497-y (PMC11210133; doi:10.1186/s12903-024-04497-y)

**Supplementary Information file: gel images of Western Blot**

**FIGURE2**

**figure2 COL1
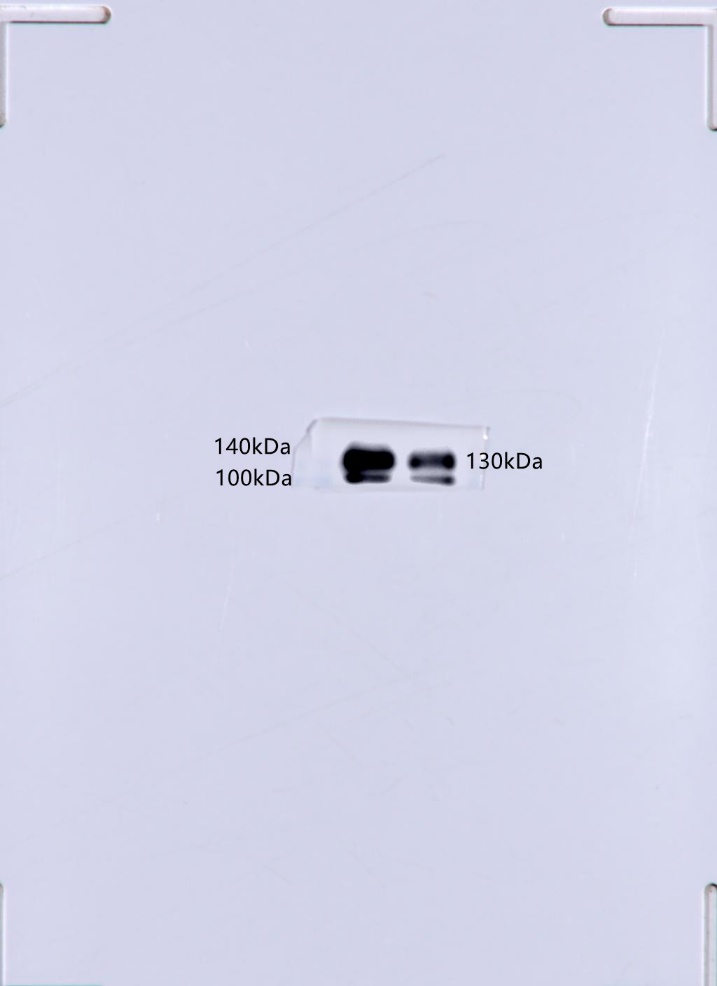
**

**figure2 RUNX2
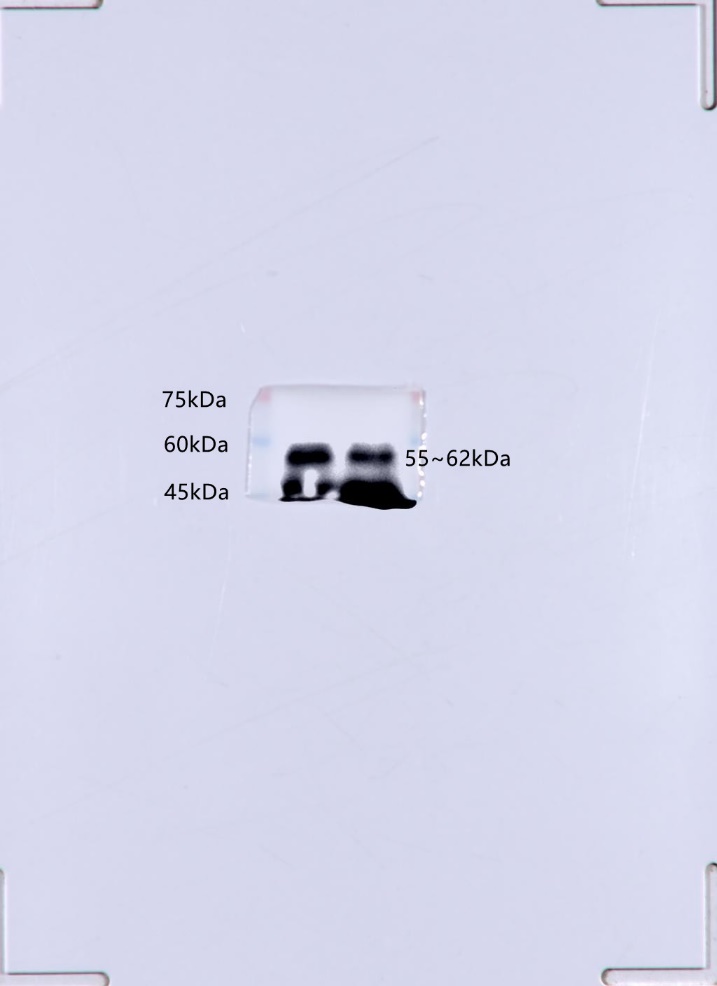
**

**figure2 GAPDH
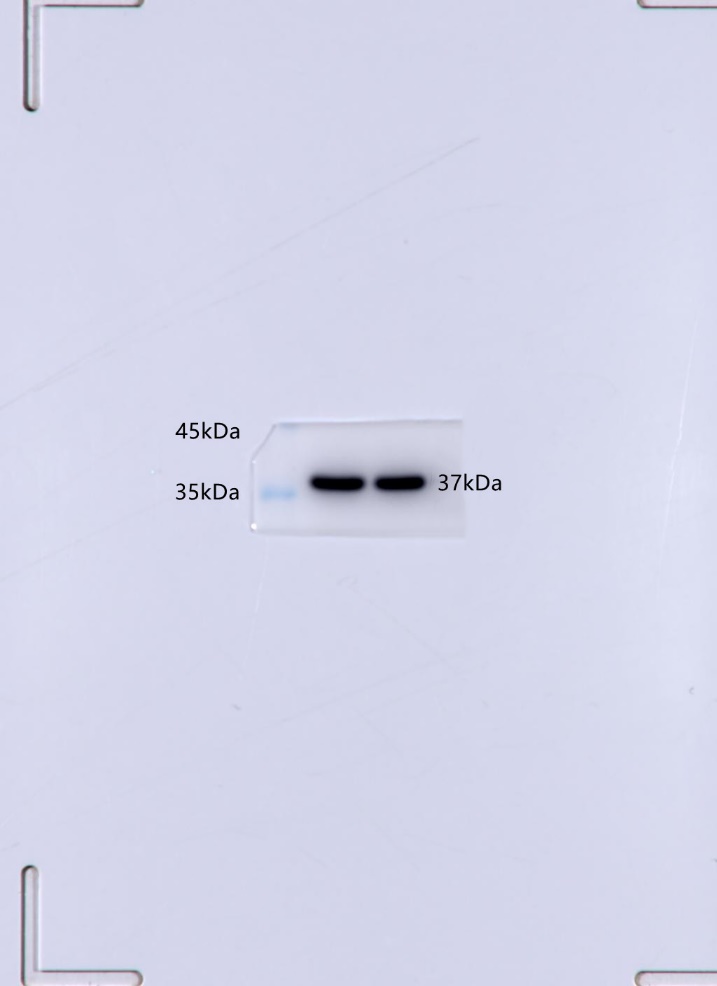
**

**FIGURE3**

**figure3 A TAZ**
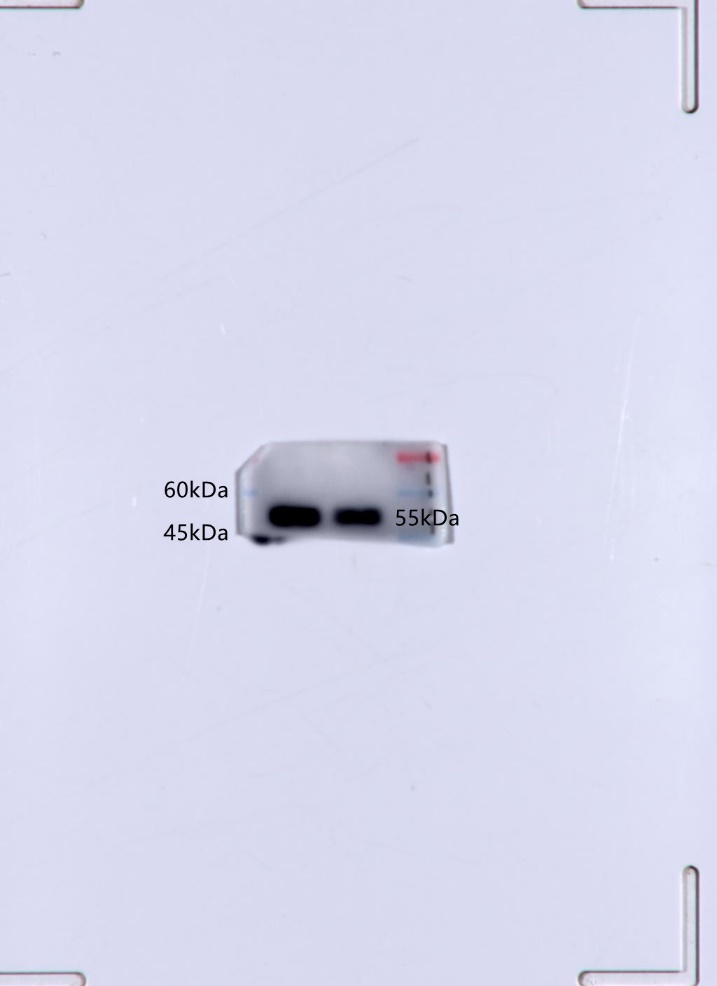


**figure3 A GAPDH**
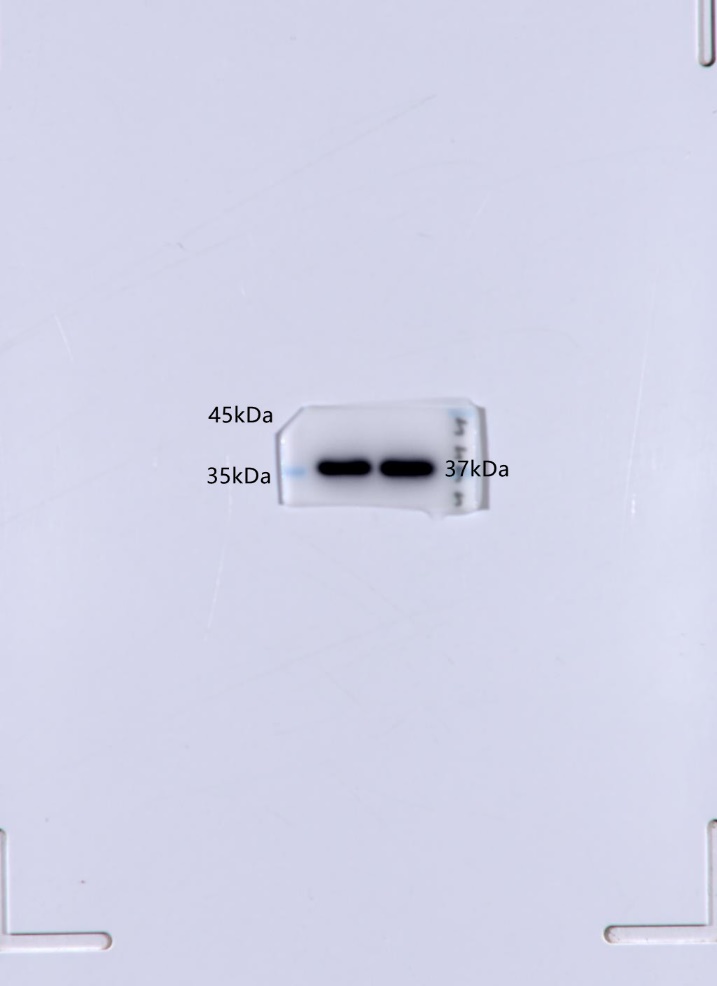


**figure3 C p-P65**
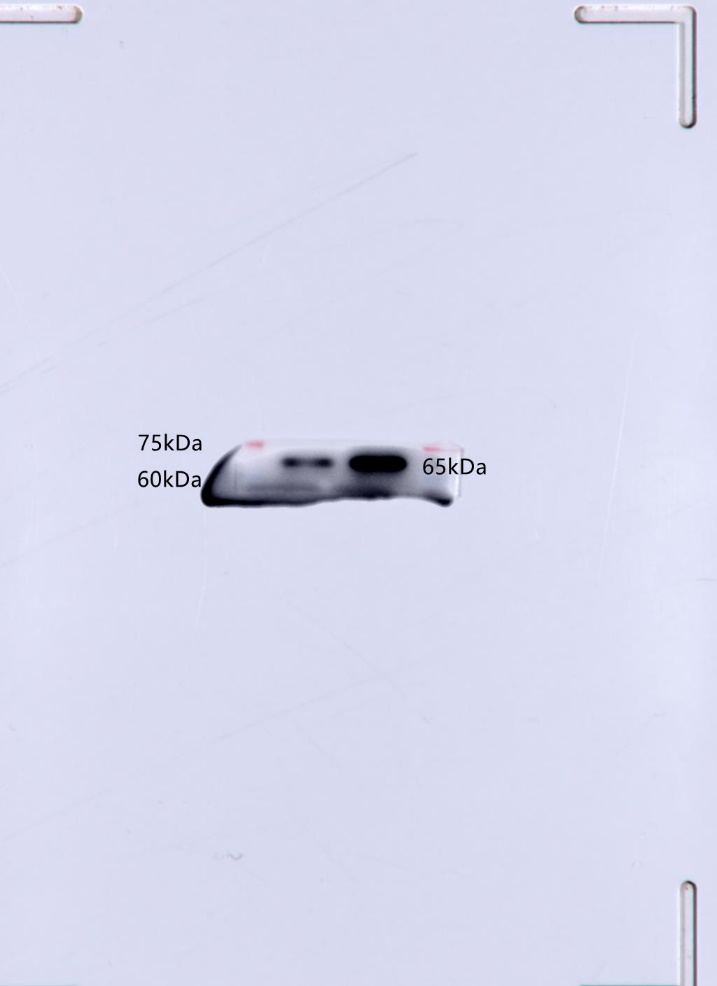


**figure3 C P65**
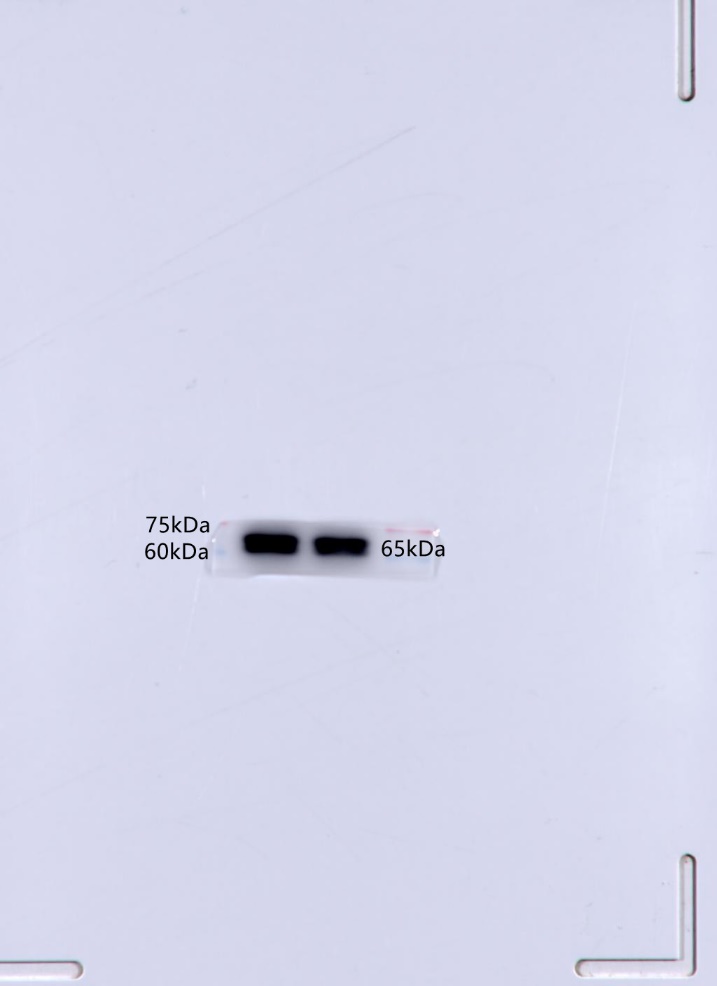


**figure3 C P-** **IκBα**
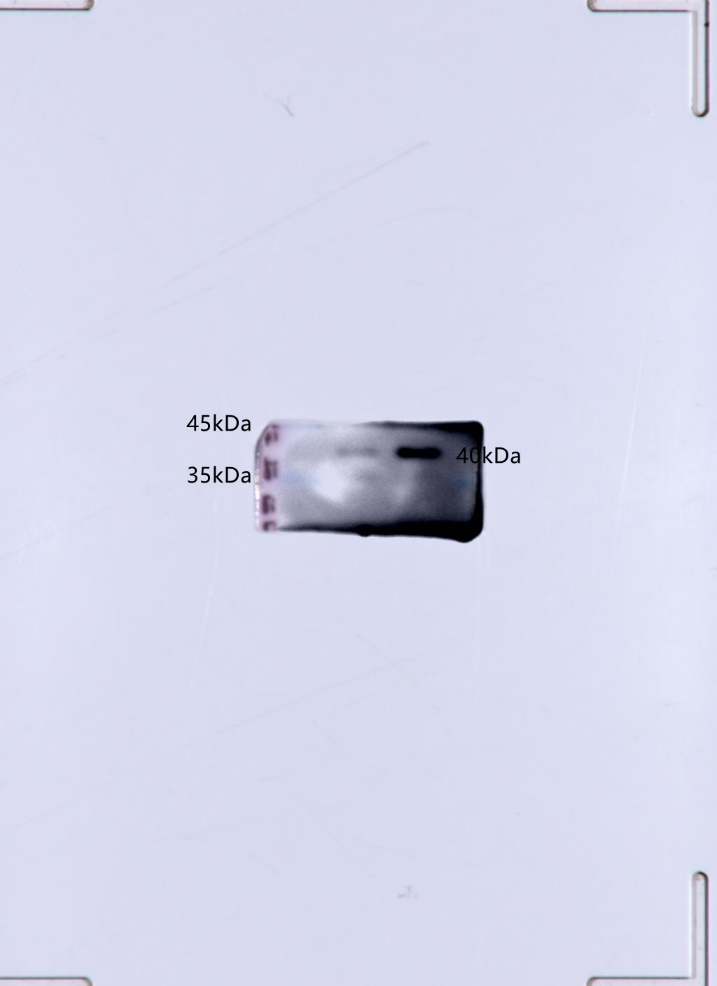


**figure3 C IκBα**
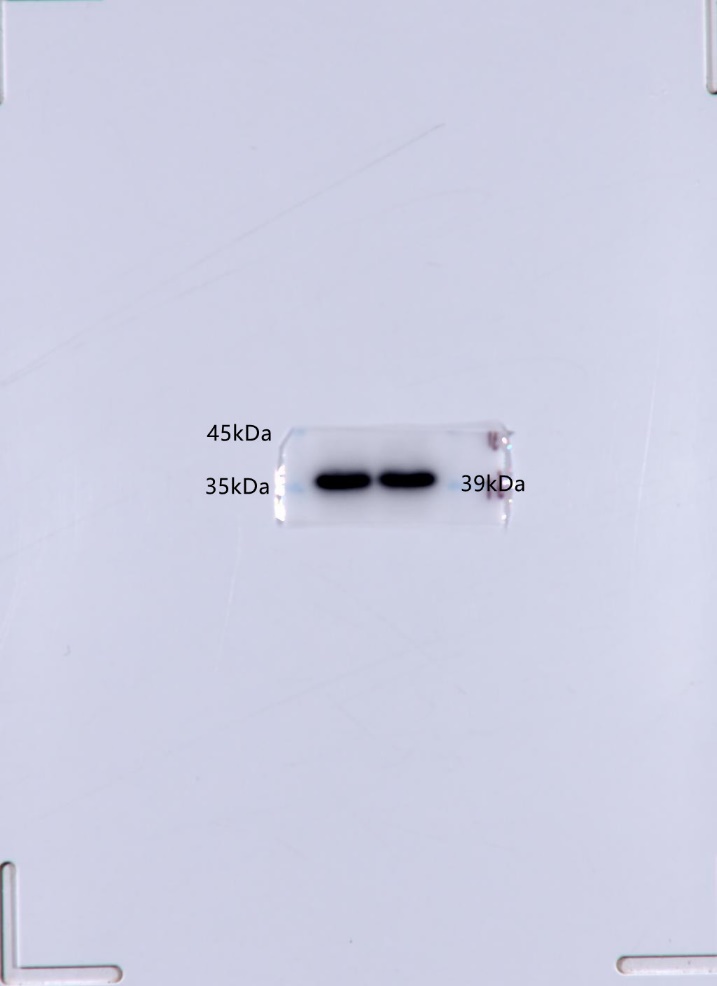


**figure3 C GAPDH**
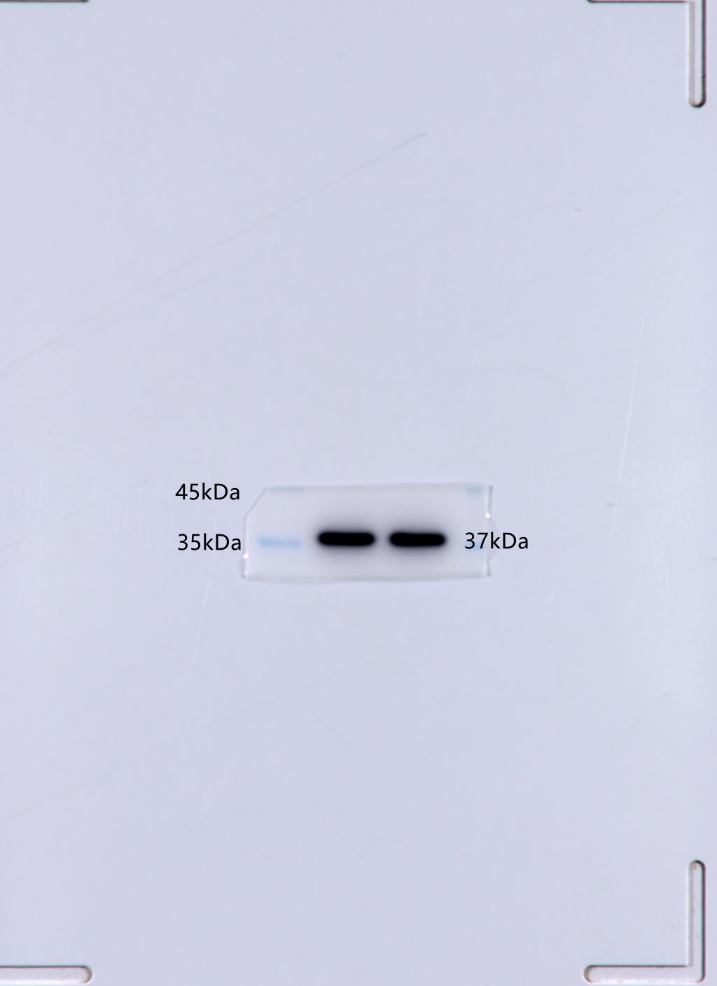


**FIGURE4**

**figure 4 B oeTAZ-TAZ**
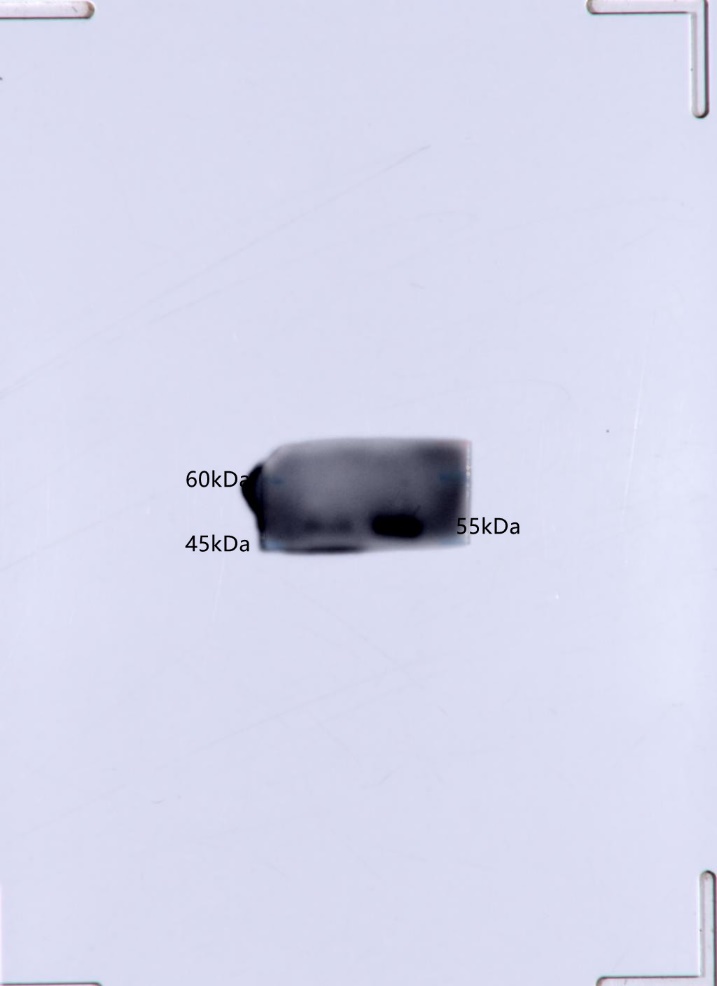


**figure 4 B oeTAZ-GAPDH**
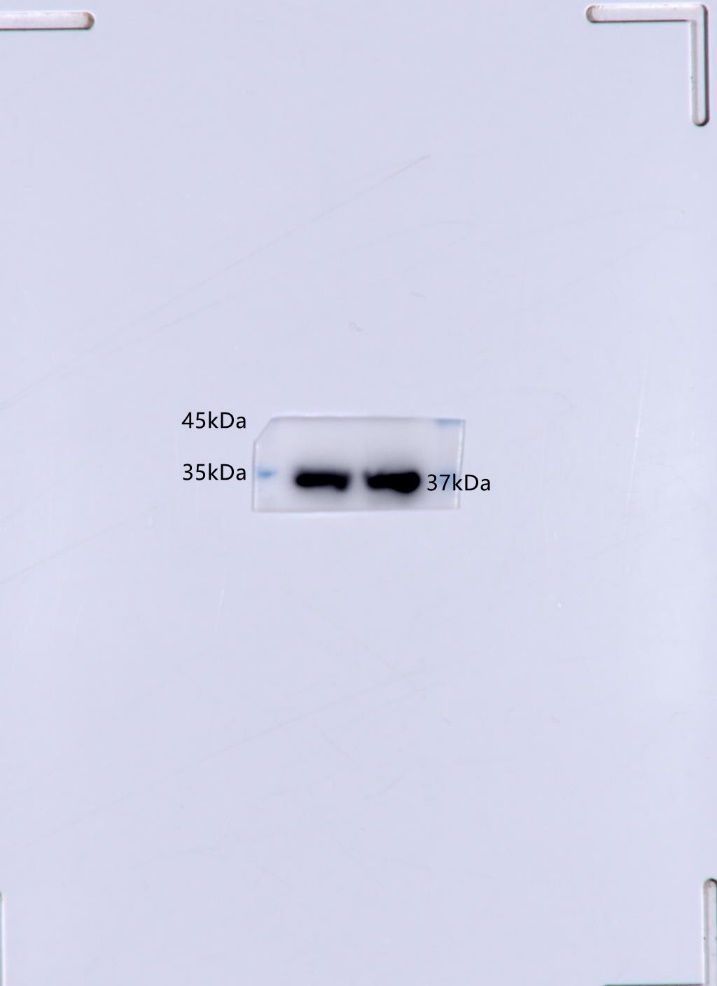


**figure 4 B shTAZ-TAZ**
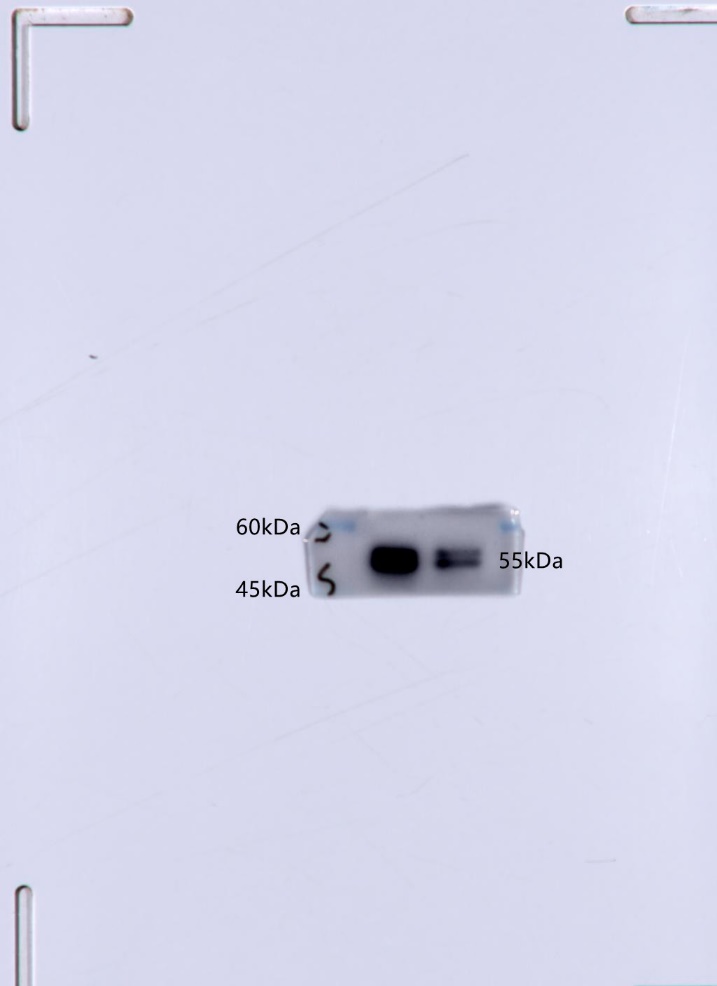


**figure 4 B shTAZ-GAPDH**
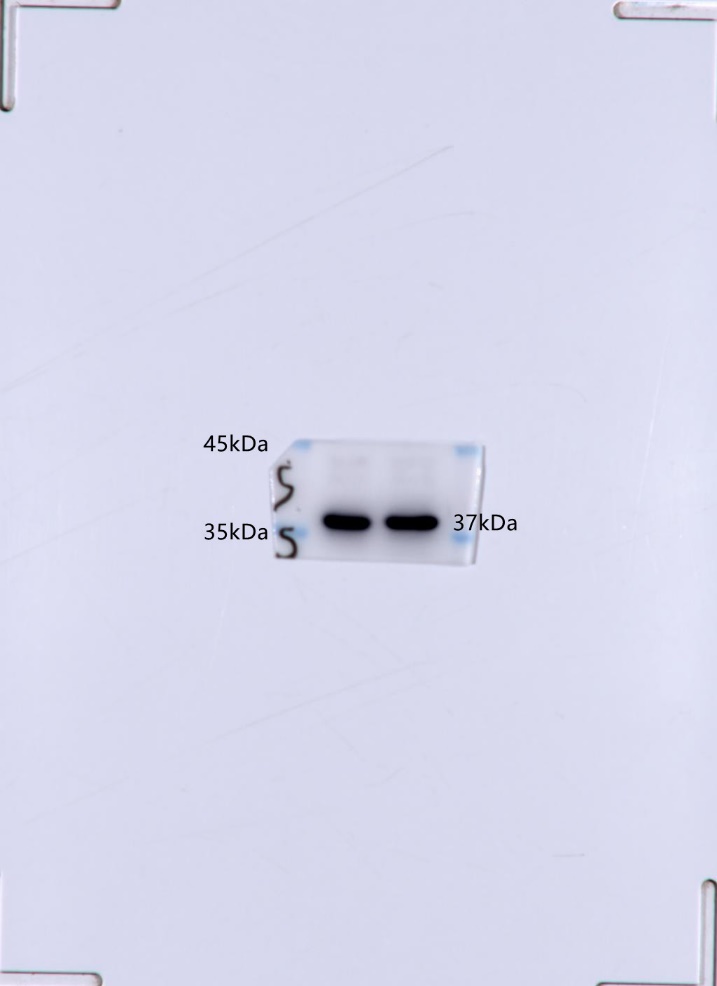


**figure 4 C RUNX2**
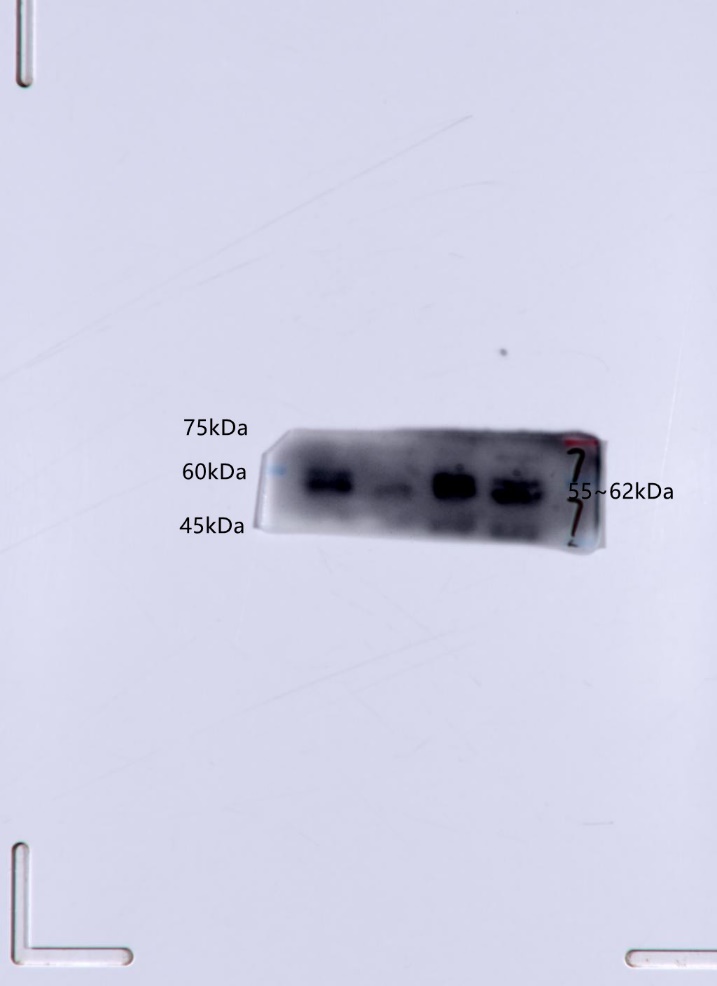


**figure 4 C ALP**
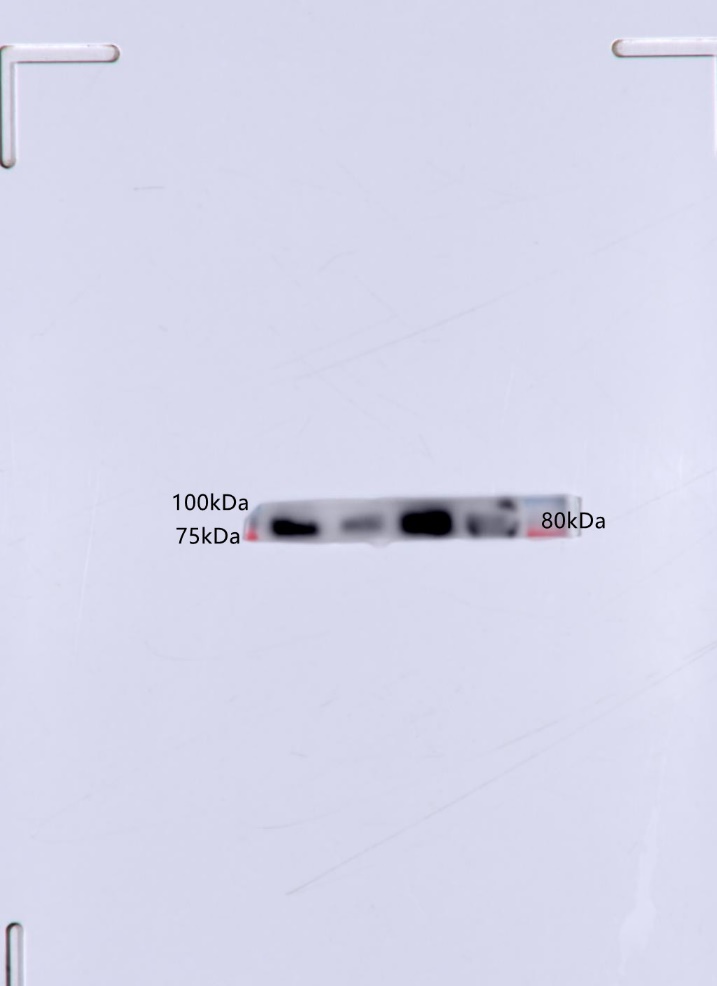


**figure 4 C GAPDH**
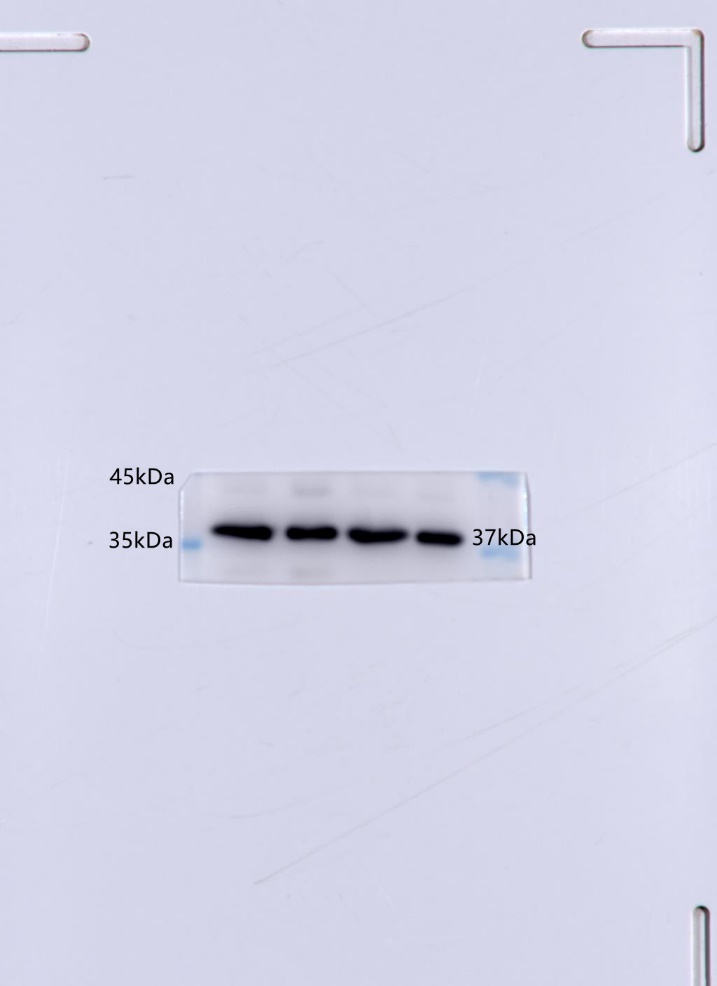


**figure 4D RUNX2**
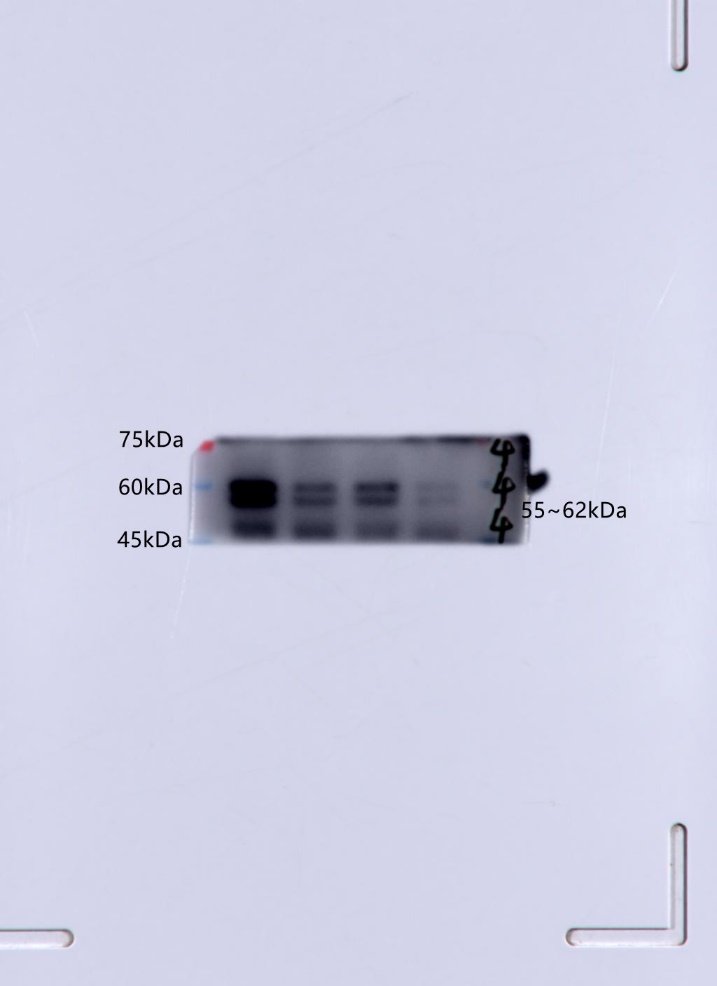


**figure 4 D COL1**
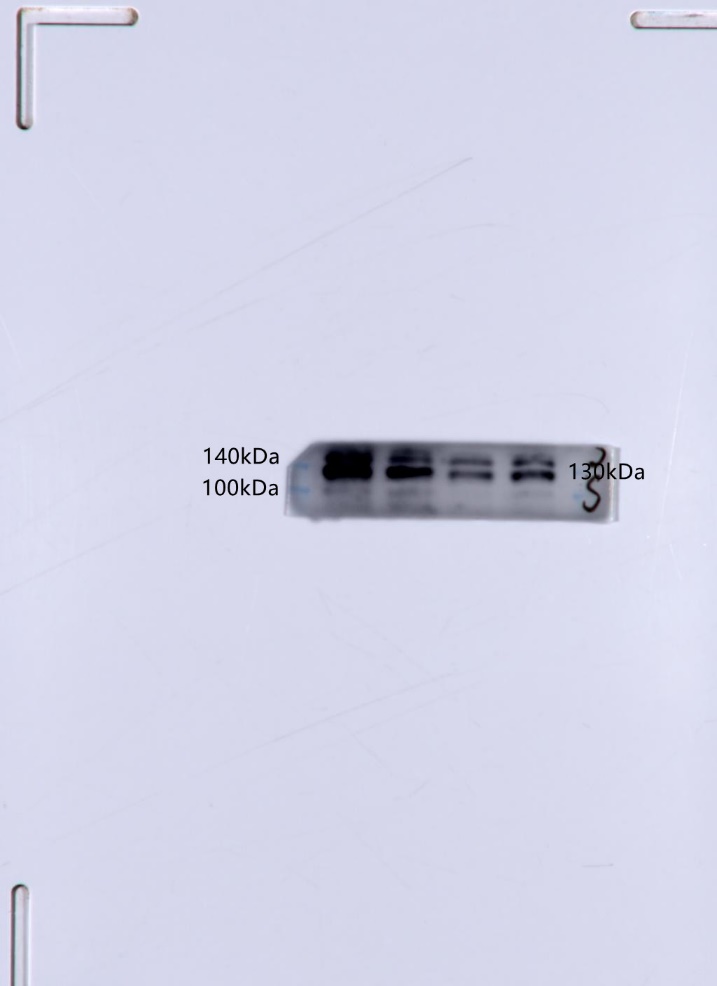


**figure 4 D-GAPDH**
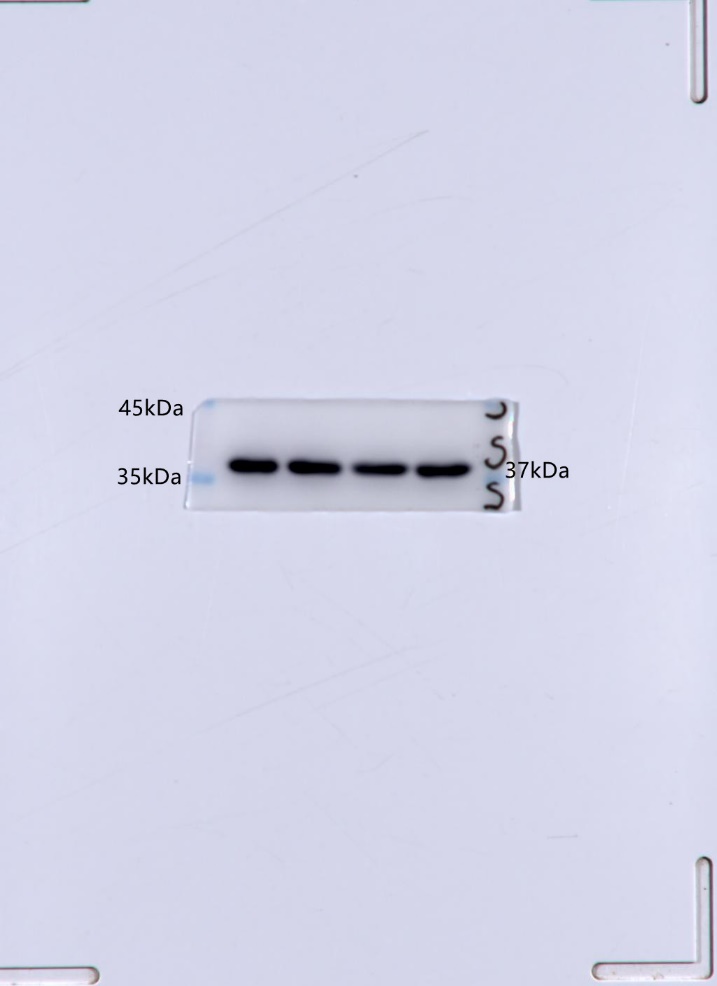


**FIGURE5**

**figure5 A p-P65**
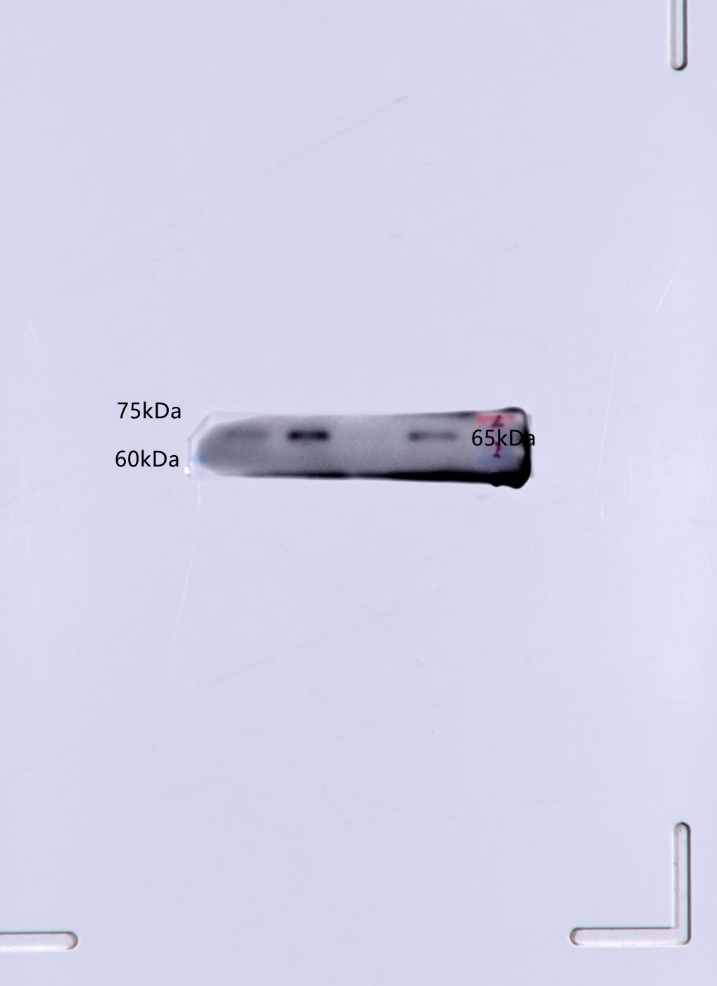


**figure5 A P65**
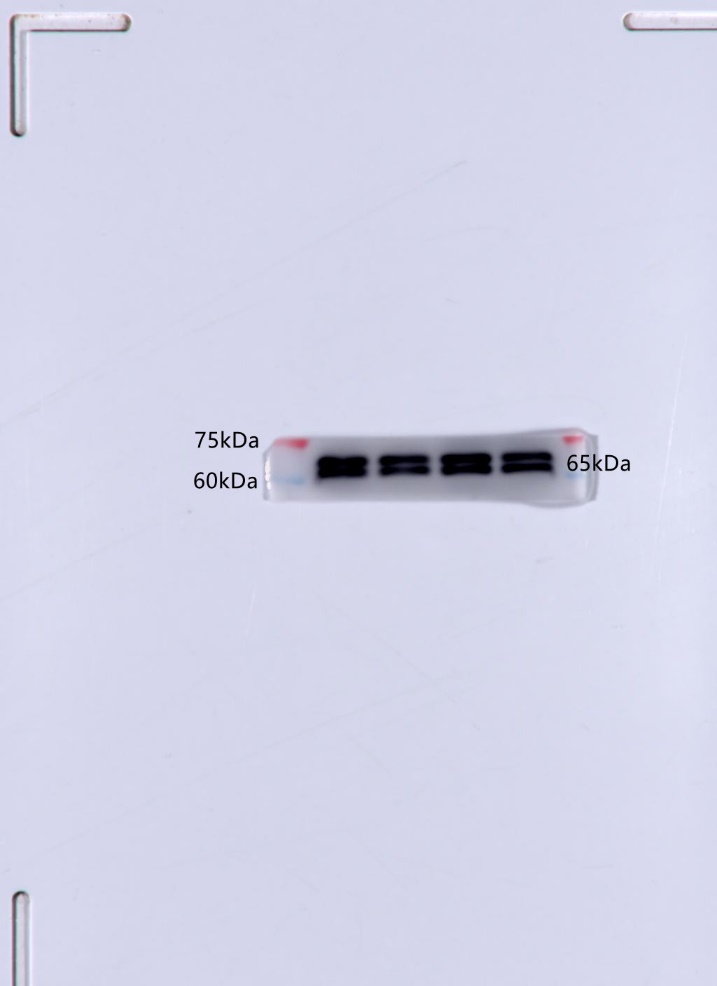


**figure5 A GAPDH1**
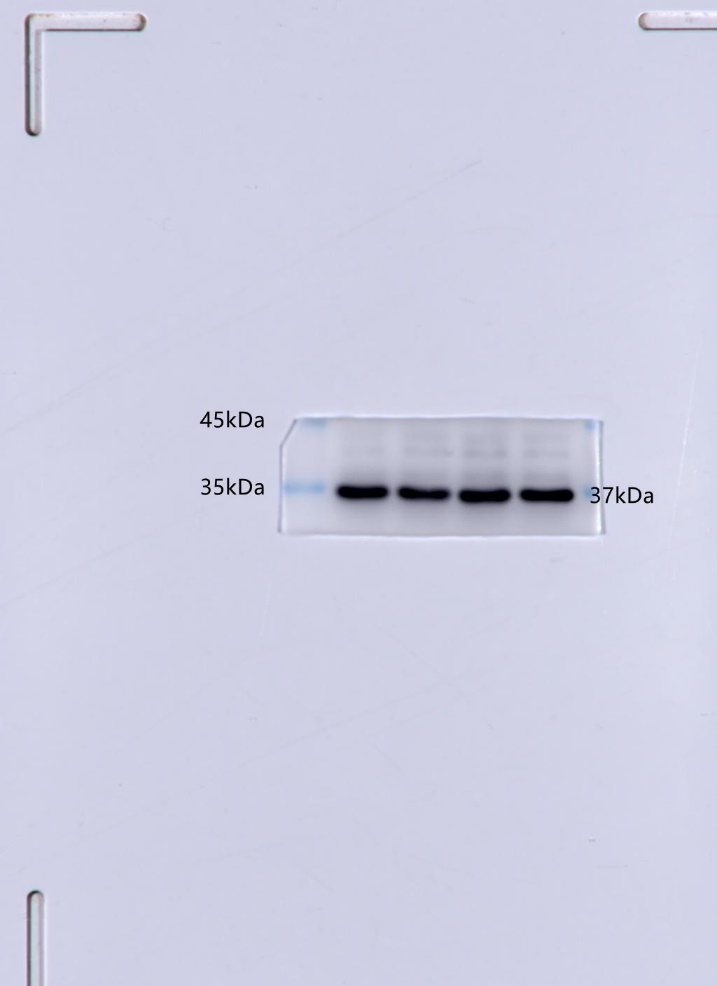


**figure5 A p- IκBα**
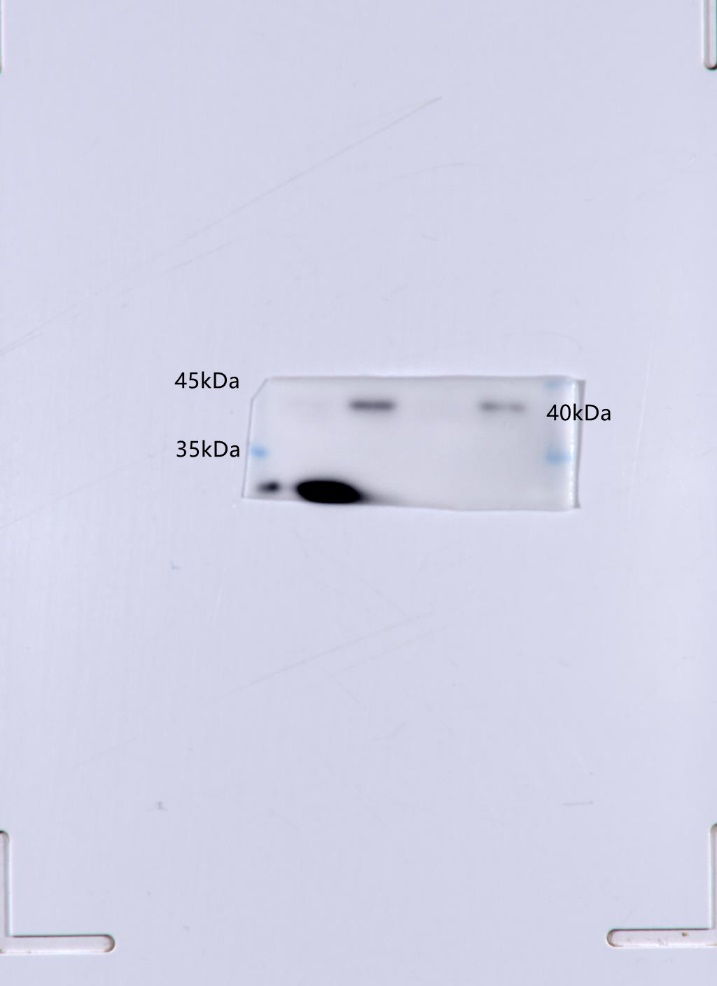


**figure5 A IκBα**
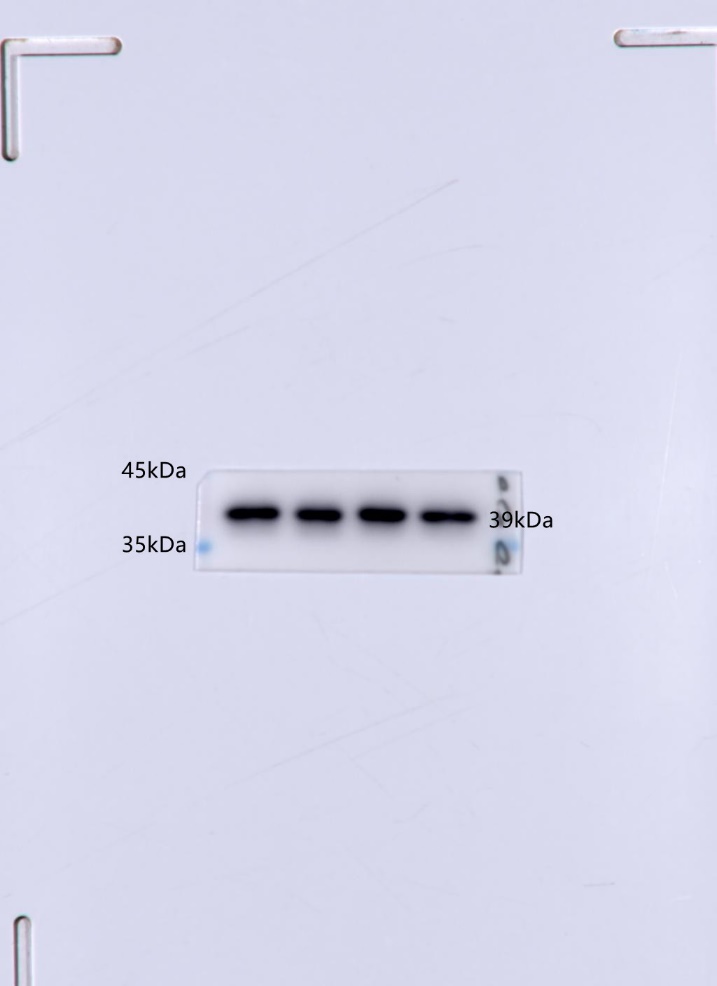


**figure5 A GAPDH2**
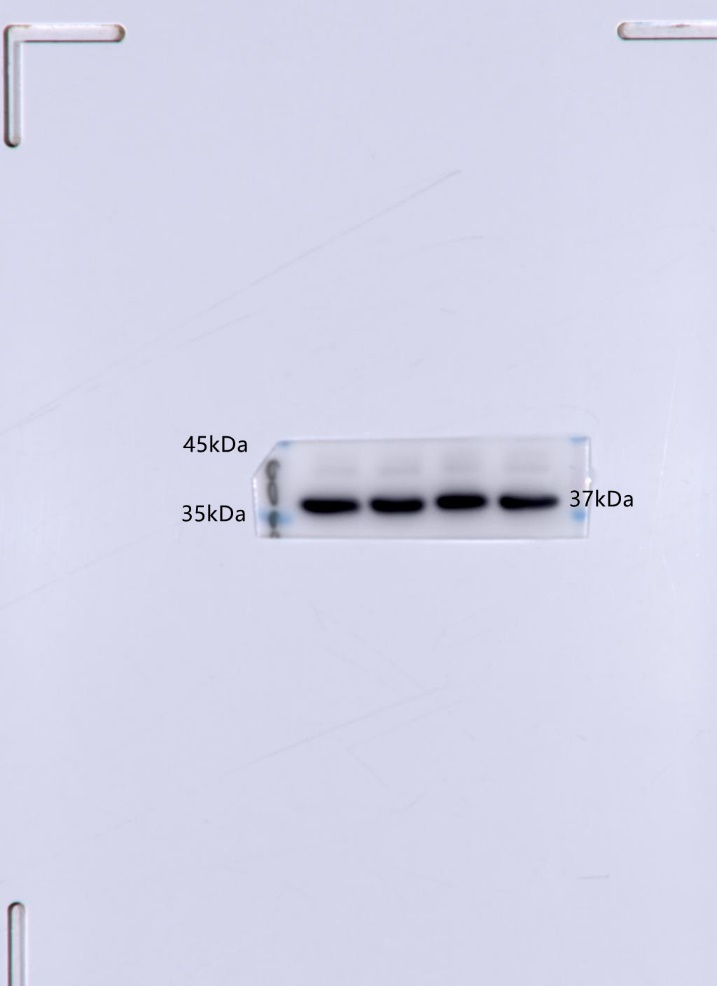


**figure5 B p-P65**
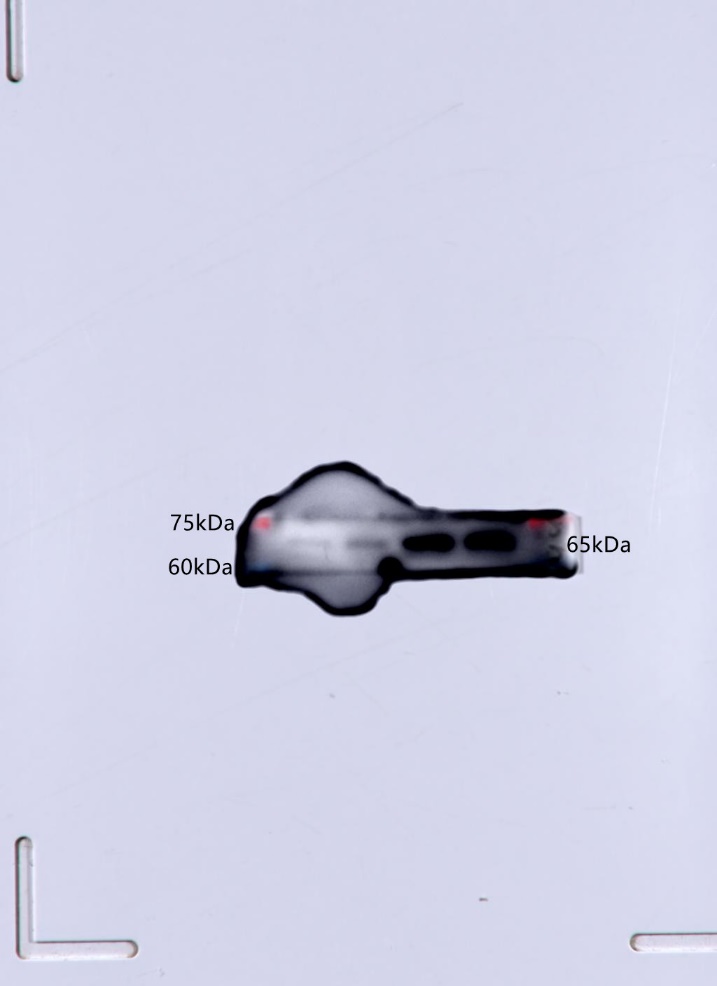


**figure5 B P65**
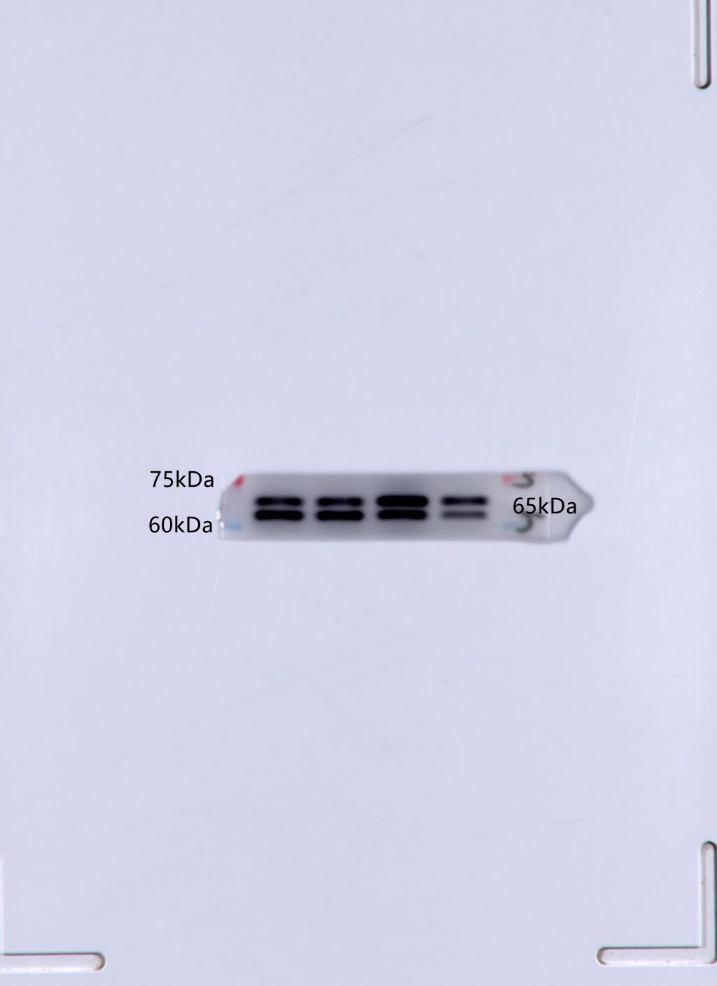


**figure5 B GAPDH1**
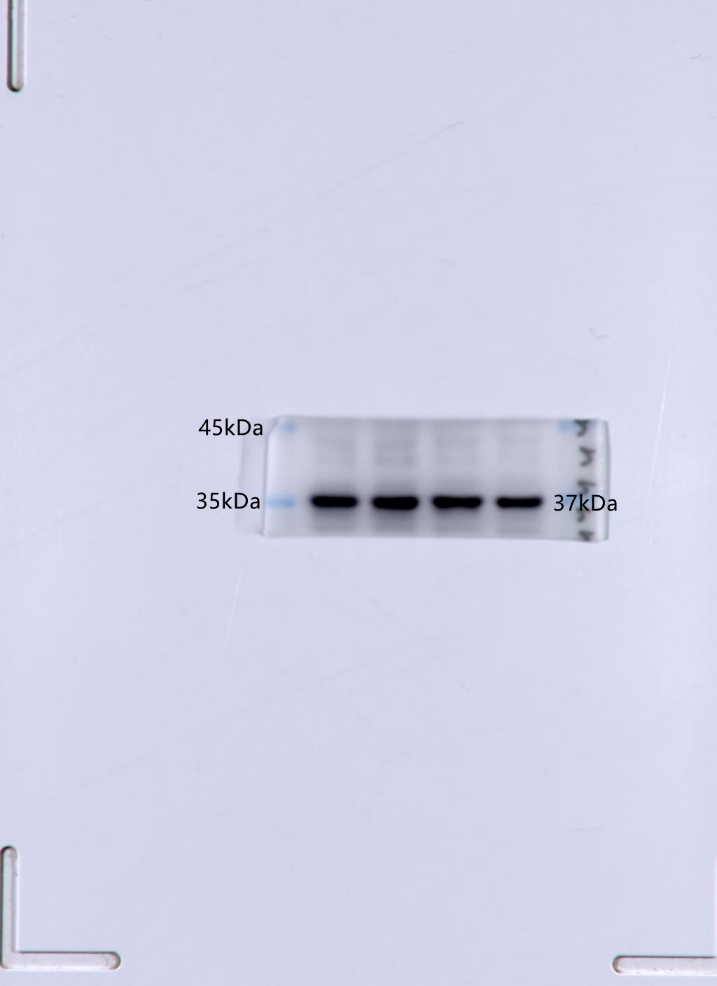


**figure5 B p- IκBα**
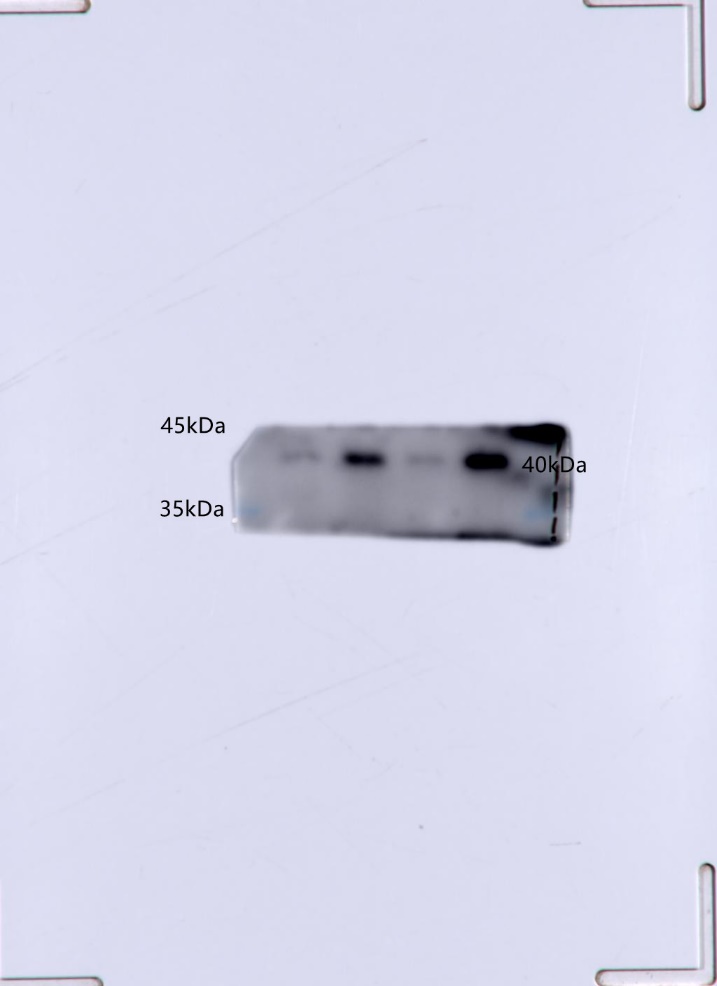


**figure5 B IκBα**
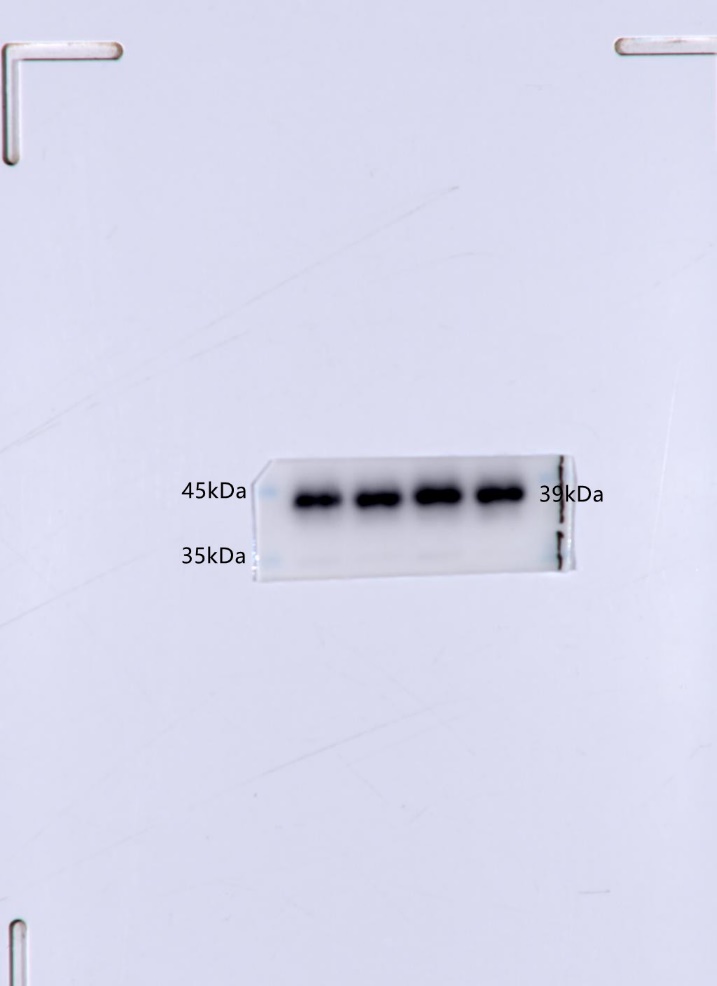


**figure5 B GAPDH2**
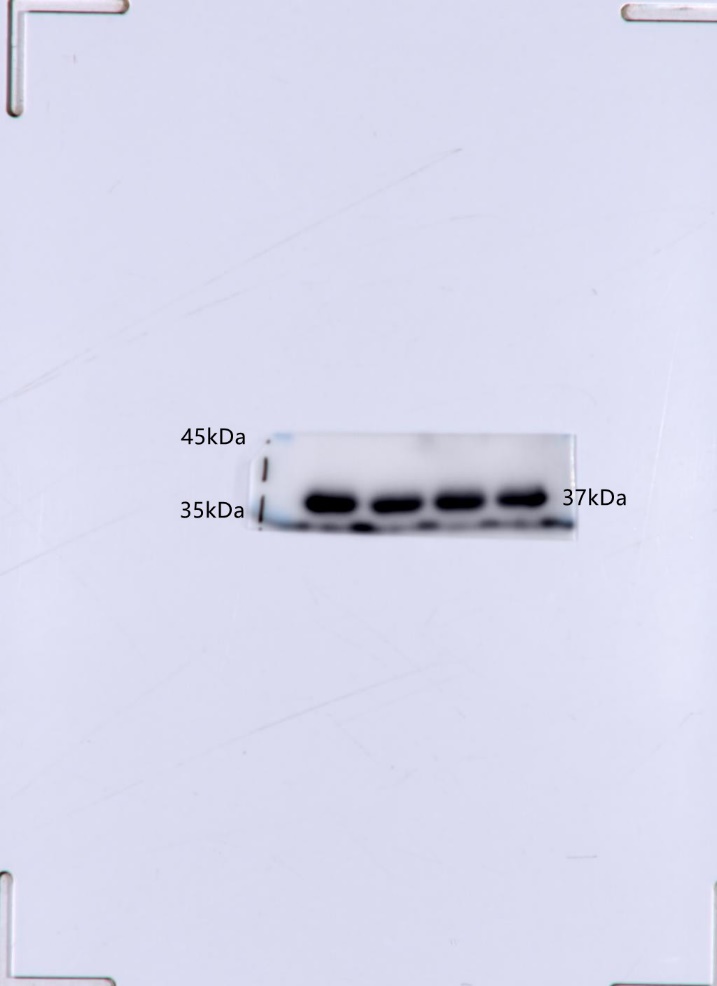

Supplement: Supplementary file 1 — Supplementary Material 1 [file 12903_2024_4497_MOESM1_ESM.docx]
